# Evaluation of Animal Models by Comparison with Human Late-Onset Alzheimer’s Disease

**DOI:** 10.1007/s12035-018-1036-6

**Published:** 2018-04-14

**Authors:** Bu-Yeo Kim, Hye-Sun Lim, Yoonju Kim, Yu Jin Kim, Imhoi Koo, Soo-Jin Jeong

**Affiliations:** 1 Herbal Medicine Research Division, Korea Institute of Oriental Medicine, 1672 Yuseong-daero, Yuseong-gu, Daejeon, 34054 Republic of Korea; 2 College of Pharmacy, Chungnam National University, 99 Daehak-ro, Yuseong-gu, Daejeon, 34134 Republic of Korea; 3 Huck Institutes of Life Sciences, Pennsylvania State University, University Park, PA 16802 USA; 4 Korean Medicine of Life Science, University of Science & Technology, 217 Gajeong-ro, Yuseong-gu, Daejeon, 34113 Republic of Korea

**Keywords:** Late-onset Alzheimer’s disease, 5×FAD, Amyloid-β, BCCAO, Microarray

## Abstract

Despite many efforts to alleviate the pathological conditions of Alzheimer’s disease (AD), effective therapeutic drugs have not been developed, mainly because of the lack of molecular information about AD and animal models. We observed the reciprocal regulation of AD-associated genes (AD genes) and their related functions. Upregulated AD genes were positioned in central regions in the protein–protein interaction network and were involved in inflammation and DNA repair pathways. Downregulated AD genes positioned in the periphery of the network were associated with metabolic pathways. Using these features of AD genes, we found that 5×FAD, amyloid β-injected mice, and rats in the initial phases after bilateral common carotid artery occlusion (BCCAO) exhibited patterns that were most similar to those of AD. In contrast, using differentially expressed genes from animal models, we observed that 3×Tg and animals in late phases of BCCAO were positioned close to AD genes.

**Electronic supplementary material:**

The online version of this article (10.1007/s12035-018-1036-6) contains supplementary material, which is available to authorized users.

## Introduction

Alzheimer’s disease (AD) is a progressive and chronic neurological disorder that causes memory impairment and cognitive deficits, primarily in the elderly. The majority of human AD cases (> 95%) are sporadic non-Mendelian patients aged ≥ 65 years [1]. The pathological characteristics of AD include the deposition of the peptide corresponding to amino acids 39–42 of amyloid beta (Aβ) and of the hyperphosphorylated forms of the microtubule-associated tau protein in the brain [2, 3]. However, the recent failures of clinical trials of novel drugs designed against Aβ have raised questions regarding the exact role of Aβ in the neurobiology of AD, although the negative results obtained in these trials do not disprove the possible effectiveness of drugs in patients with early onset AD [4, 5]. Moreover, genome-wide association studies (GWASs) based on a large population of patients with late-onset AD identified several genetic loci, the most prominent of which are not directly connected with Aβ and tau physiology [6, 7]. This genetic difference between familial AD and sporadic AD strongly suggests the involvement of separate processes in the development of the two types of AD.

In addition to the clinical effectiveness of drugs, the manner in which the animal models that are used in preclinical trials can simulate human AD should also be considered. Many AD animal models, including genetically and nongenetically engineered animals, have been developed in the last two decades, and their pathophysiological traits have been identified. Genetically modified transgenic models focus primarily on mutated genes that were identified in patients with familial AD, which include APP, MAPT, PSEN1, and PSEN2 [8, 9]. These genes have been used alone or in combination to produce animal models with cognitive impairment. The functions of these genes are mainly implicated directly in Aβ and/or tau physiology, thus supporting the contention that the accumulation of extracellular Aβ and/or intracellular tau could lead to cognitive impairment in humans. In addition to transgenic models of AD, pharmacological models that reproduce transiently the symptomatic features of AD have also been used widely, including the scopolamine-induced [10] and streptozotocin-induced rodent models [11]. Although transgenic and nontransgenic animal models are valuable tools in the identification of the mechanisms of AD pathologies, these models do not reproduce all of the abnormalities observed in human AD. In particular, most transgenic models do not replicate the molecular features of sporadic AD, which is not caused by mutations in genes directly associated with Aβ physiology, as demonstrated by a massive genome-wide analysis [6]. These previous reports strongly suggest that, despite the pathological similarities between animal models of AD and human AD, such as memory loss and cognitive impairment, the molecular processes resulting in those pathological conditions may be different in each model, depending on genetic or other causative factors.

Therefore, the identification of the molecular features of AD and the evaluation of animal models of AD in terms of these features are urgently required, to obtain information about which model is optimal for the development of novel anti-AD drugs. In the present study, we reanalyzed genes that were shown to be associated with late-onset AD in external sources of microarrays and evaluated their biological roles using function- and network-based approaches. We observed that the reciprocal regulation of genes and their associated functions in AD depended on their expression levels. Using these findings, the similarities between the features of various AD models were measured and compared with those of human AD.

## Materials and Methods

### Animal Experiments

Transgenic mouse models, including those overexpressing the mutated human NCSTN gene (KNL-HYD-TG0610, 10-month-old male) [12], the mutated human PSEN2 gene (KNL-HYD-TG0606, 12-month-old male) [13], and the wild-type human MAPT gene (KNL-HYD-TG0601, 12-month-old male) [14], were provided by the National Institute of Food and Drug Safety Evaluation of Korea (Cheongju, Korea). The C57BL/6N background strain was used as the normal control for KNL-HYD-TG0610 and KNL-HYD-TG0606 animals. C56BL/6N was used as the normal control for the MAPT model (KNL-HYD-TG0601).

To induce cognitive impairment using external chemicals, scopolamine, Aβ, or streptozotocin were administered to ICR male mice (7 weeks of age) purchased from Daehan Biolink Co. Ltd. (Eumseong, Korea). Scopolamine (Sigma-Aldrich, St. Louis, MO, USA) was administered by intraperitoneal (i.p.) injection at a dose of 1 mg/kg 30 min before the isolation of brain tissues. The amyloid-β_1–42_ peptide (AnaSpec, Inc., Fremont, CA, USA) was dissolved in a PBS solution (137 mM NaCl, 10 mM Na_2_HPO_4_, 1.8 mM KH_2_PO_4_, and 2.7 mM KCl at pH 7.5) at a concentration of 1 mM/μL and aggregated by incubation at 37 °C for 7 days prior to use. Aβ (5 or 10 μM) or streptozotocin (2.5 or 3 mg/kg; Sigma-Aldrich) was injected into the intracerebroventricular (i.c.v.) region. During this process, ICR mice were positioned in a stereotaxic frame and a midline sagittal incision was made on the scalp. Holes were drilled in the skull over the lateral ventricles using the following stereotaxic coordinates: − 0.46 mm anteroposterior, 1 mm mediolateral, and 2 mm dorsoventral. All injections were performed using a 5 μL Hamilton syringe equipped with a 26S-gauge needle. Aβ or streptozotocin were injected at the rate of 1 μL/min, to a final volume of 3 μL. Brain tissues were prepared 14 days after i.c.v. injection of Aβ or streptozotocin.

Twelve-week-old male Wistar rats from OrientBio Inc. (Seongnam, Korea) were used for the surgical induction of bilateral common carotid artery occlusion (BCCAO). Rats were randomly divided into six groups (sham and 21, 35, 45, 55, and 70 days after BCCAO). BCCAO was induced as described previously, with some modifications [15, 16]. Briefly, the rats were anesthetized using 5% isoflurane, and the bilateral common carotid arteries were tightly double ligated with silk sutures. For the control sham group, the same procedure was performed without BCCAO. For the study of aging, male C57BL/6 mice aged 1.5, 4, 9, 17, and 22 months and raised in a specific pathogen-free facility were obtained from the Korea Basic Science Institute (Gwangju, Korea). Six-month-old male 5×FAD mice (Tg(APPSwFlLon,PSEN1*M146L*L286V)6799Vas) were obtained from the Jackson Laboratory (Bar Harbor, Maine, USA). The C57BL/6N background strain was used as the normal control for 5×FAD animals. All animals were housed individually in polycarbonate cages at a controlled temperature of 23 ± 3 °C with 60% humidity and a 12-h light/dark cycle. Standard chow diet (catalog no. 6112, Central Laboratory Animal Inc., Seoul, Korea) and water were provided ad libitum throughout the experiment. All mice were fasted overnight and anesthetized using 5% isoflurane before sacrifice. Every effort was made to minimize pain during the experimental period. For biological analysis, brain regions were dissected and frozen at − 80 °C until microarray analysis. All experimental procedures were approved by the Institutional Animal Care and Use Committee of the Korea Institute of Oriental Medicine and were performed in strict accordance with the recommendations of the Guide for the Care and Use of Laboratory Animals of the Korea Institute of Oriental Medicine.

### Microarray Experiment

Total RNAs were purified from each brain region using an RNeasy kit according to the manufacturer’s instructions (Qiagen, Hilden, Germany). After checking the quality of the RNA using a Bioanalyzer 2100 RNA Nano Kit (Agilent Technologies, Santa Clara, CA, USA), only samples with an RNA integrity number (RIN) > 7.0 were included in the microarray analysis. To minimize the effects of individual variability, RNAs isolated from tissues of three animals were pooled in equal amounts, followed by amplification and labeling using a Low RNA Input Linear Amplification Kit PLUS, according to the manufacturer’s instructions (Agilent Technologies). Finally, labeled RNAs were hybridized to a microarray (Agilent Mouse Whole Genome 44K for mouse brain tissues and Rat Whole Genome 44K for rat brain tissues) using the Gene Expression Hybridization Kit, according to the manufacturer’s instructions (Agilent Technologies). The arrays were then scanned using an Agilent DNA Microarray Scanner, and the raw signal intensities from the scanned image were extracted using the Agilent Feature Extraction Software (Agilent Technologies). Array information was deposited at the Gene Expression Omnibus (http://www.ncbi.nlm.nih.gov/geo) public site under ID numbers GSE44289 and GSE104031, respectively.

### Microarray Analyses

Probes on arrays with signal intensities that were 1.4-fold higher than the local background were selected exclusively, as described previously [17, 18], and were then normalized using the quantile method, which can adjust the variability within and between samples [19]. Hierarchical clustering based on the expression levels of genes was performed using the Gene Cluster 3.0 program and visualized using the Java TreeView program [20]. Mouse and rat gene symbols were compared with human gene symbols based on the orthology database maintained by the Jackson Laboratory (http://www.informatics.jax.org).

### Transcription Factor Binding Site Analysis

Frequency measurement of binding sites for transcription factors in the promoter region of genes was conducted by implementing the MotifDb R package (version 1.18.0, https://www.bioconductor.org/packages/release/bioc/html/MotifDb.html) [21]. A total of 459 position weight matrices were used as binding sites for each human transcription factor from the JASPAR database (http://jaspar.genereg.net) [22]. The nucleotide sequences of the promoter region of genes covering 2000 bp upstream to 500 bp downstream from the transcription start site were obtained from the human full genome sequences provided by the University of California, Santa Cruz (UCSC hg38 version). The putative transcription factor binding sites (TFBS) were predicted via sequence matching of the promoter regions of genes with the position weight matrix of each transcription factor using the matchPWM algorithm with a minimum score of 0.9 for counting a match [23]. The resultant matrices of the TFBS frequency from genes were clustered to measure similarity between genes by implementing Jaccard’s algorithm, which minimizes the effects of the absence of TFBS at each gene promoter [24, 25].

### Gene Ontology and Pathway Analyses

A simple enrichment analysis for gene ontology (GO) terms and pathways was performed using the Functional Annotation Tool of Database for Annotation, Visualization and Integrated Discovery (DAVID) [26], in which a modified Fisher’s exact test was used and false discovery rate (FDR) was calculated using the Benjamini–Hochberg procedure, as a measurement of statistical significance. Based on the enriched GO terms obtained from the DAVID analysis, nonredundant GO terms and their connections based on semantic similarity were obtained using the Reduce and Visualize Gene Ontology program (REVIGO) [27].

A more systematic functional enrichment analysis was performed using gene set enrichment analysis (GSEA), which calculates the enrichment score by measuring the correlation between gene sets and phenotypic class using all genes included in each gene set [28]. After a random permutation of 1000, statistical significance was presented as nominal *p* values and FDR *q* values. As another measurement of pathway enrichment using the expression values of all genes included in each pathway, pathway activity was examined by calculating the cumulative effect of the expression of all genes in each pathway [15, 18]. In brief, the log-transformed expressional ratios of genes relative to the normal control in each pathway were added linearly with a weight of − 1 for genes that acted as repressors, which were defined as proteins that inhibited the process of signal transduction of the pathway. The measured value of pathway activity was normalized by dividing it by the size of the pathway. Statistical significance was calculated by comparing the measured values with those obtained from a random permutation of 1000. The pathway information was obtained from the Kyoto Encyclopedia of Genes and Genomes database (KEGG, http://www.genome.jp/kegg/).

### Node Degree Distribution in the Protein–Protein Interaction Network

The distribution of the node degrees of genes was measured in the protein–protein interaction (PPI) network, which was constructed using information from low-throughput experiments obtained from the BioGRID database (version 3.4.149) [29]. After measuring the degrees of node included in a set of genes, the proportion of those genes in a whole network was calculated by increasing degree values. When the number of genes at a specific degree value was < 10, we added those genes to the genes measured at the next degree value, to reduce fluctuations resulting from small numbers.

### Functional Network

Functional connections between GO terms were constructed by implementing the ClueGO Cytoscape plugin application (http://www.ici.upmc.fr/cluego/) [30]. Input genes were queried into ClueGO using the following settings: Benjamin–Hochberg FDR < 0.01, kappa score > 0.4, and GO term fusion (to delete duplicated GO terms).

### Distances in the PPI Network

To measure distances between two gene sets, each gene was mapped onto the PPI network. Subsequently, the shortest paths between two genes from each gene set were measured using the igraph R package (version 1.0.10) [31]. After iteration of this process for all pairs of genes from two gene sets, we obtained a matrix of shortest paths. The averaged value from the matrix of shortest paths was presented as the distance between two sets of genes. Only sets of genes with a presence in the PPI network > 50% were included. Identical processes were carried out using randomly selected gene sets composed of a varying number of genes, to adjust for the effect of the size of gene sets (number of genes included in each gene set) on distances.

### Public Microarray Data

Eight public microarray datasets (GSE15222, GSE44772, GSE33000, GSE48350, GSE5281, GSE53890, GSE30272, and GSE1572), for the selection of AD- or age-associated genes, and four microarray datasets (GSE31372, GSE36981, GSE60911, and GSE80465), for animal model research, were used in the present analysis. Information on all datasets was deposited in the Gene Expression Omnibus (http://www.ncbi.nlm.nih.gov/geo). The characteristics of datasets are summarized in Supplementary Table 1. For datasets that used two-color microarray systems, including GSE44772, GSE33000, and GSE30272, background-subtracted raw intensity values were normalized using the quantile method [19]. For a one-color microarray system, raw CEL files were imported into the affy R package (version 1.54.0) and normalized using the Robust Multiarray Average (RMA) algorithm [32]. The GSE48350, GSE5281, and GSE53890 datasets were merged. To adjust for the batch effects resulting from the use of various microarray datasets, we applied empirical Bayesian methods by implementing the ComBat function in the sva R package (version 3.24.4) [33]. We adjusted for the effects of age and sex on the expression levels of genes using robust regression.

Genes that were differentially expressed between AD and nondemented individuals and genes that were associated with age were selected using significance analysis of microarrays (SAM) under the two-class response type or quantitative response type options, respectively, with a random permutation of 1000 [34]. The threshold values for gene selection were determined to be FDR < 0.01 for each microarray dataset. For GSE33000 (dataset 1) and GSE44772 (dataset 2), threshold values were set more stringently at FDR < 0.001, to reduce the number of selected genes.

### Selection of Animal Models

The significance of the measurement of the similarity between animal models and human AD was examined using a permutation-based approach. For gene expression and pathway activity analyses, equal-sized genes or pathways corresponding to those isolated from human AD were randomly selected for each animal model and their expression levels or the activities of pathways were measured. For network analysis using differentially expressed genes (DEGs) from each animal model, we also randomly selected equal-sized genes corresponding to the size of DEGs and measured distances in the PPI network. This process was iterated 1000 times. From the distribution of the averaged expression levels or activities of pathways corresponding to randomly selected genes and pathways, the proportion of random sets of genes and pathways that exhibited values of expression or activity that were greater than the original values was calculated and used in statistical analyses. The threshold values of significance were set at 0.01 and 0.05 for strong and weak correlation, respectively. Animal models that satisfied this condition in both directions (upregulation and downregulation) were selected as significant models.

#### Data Availability

All other data generated or analyzed during this study are included in this published article and its supplementary information files.

## Results

**Fig. 1:**
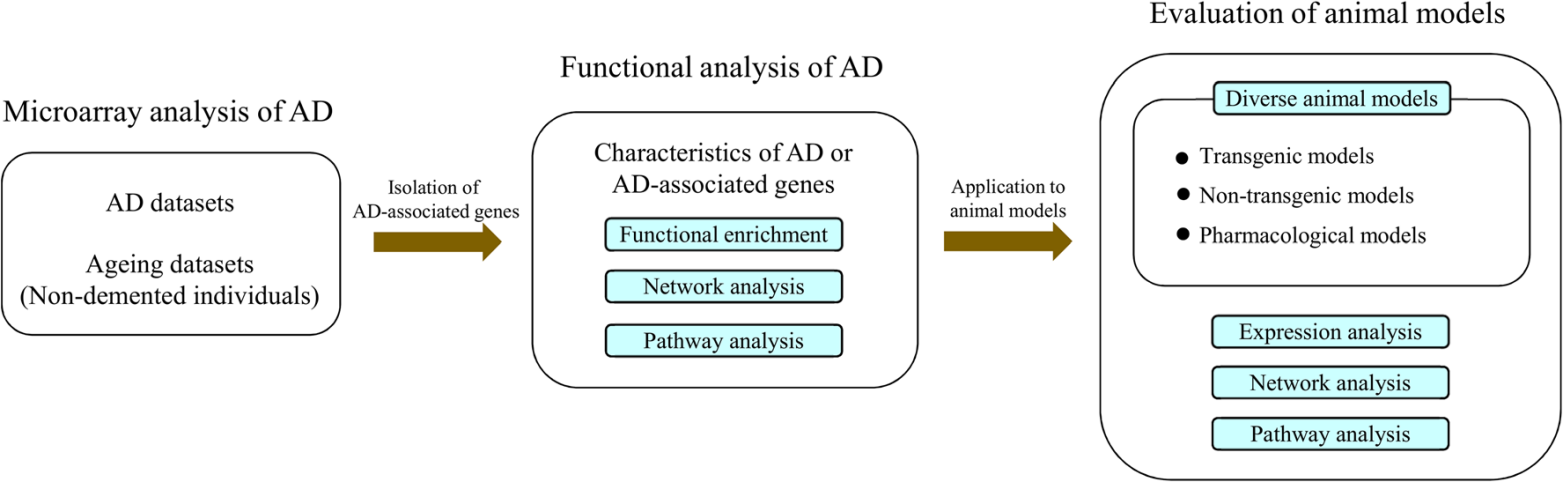
Schematic diagram of the overall experimental procedure. Microarray datasets composed of patients with AD and nondemented individuals were analyzed, to isolate AD-associated genes (AD genes). The functional characteristics of AD or AD genes were identified via functional enrichment, pathway analysis, and network analysis. These characteristics were used to evaluate various animal models of AD, including transgenic, nontransgenic, and pharmacological models. Similarities between animal models and human AD were measured using the expression levels of AD genes, pathway activities, and distances in the PPI network

The overall flow of the experiments performed here is shown in Fig. 1. Briefly, multiple microarray datasets composed of AD cases and nondemented individuals from public archives were introduced into our analysis platform. Subsequently, AD-associated genes (AD genes) were isolated from these datasets, and their biological features were analyzed. As a final step, the molecular characteristics of AD or AD genes were applied to various animal models, to measure the correlations between each model and human AD. The animal models analyzed included transgenic, nontransgenic, and pharmacological models.

### Correlation Patterns Based on the Expression Levels of Genes Among Samples

**Fig. 2:**
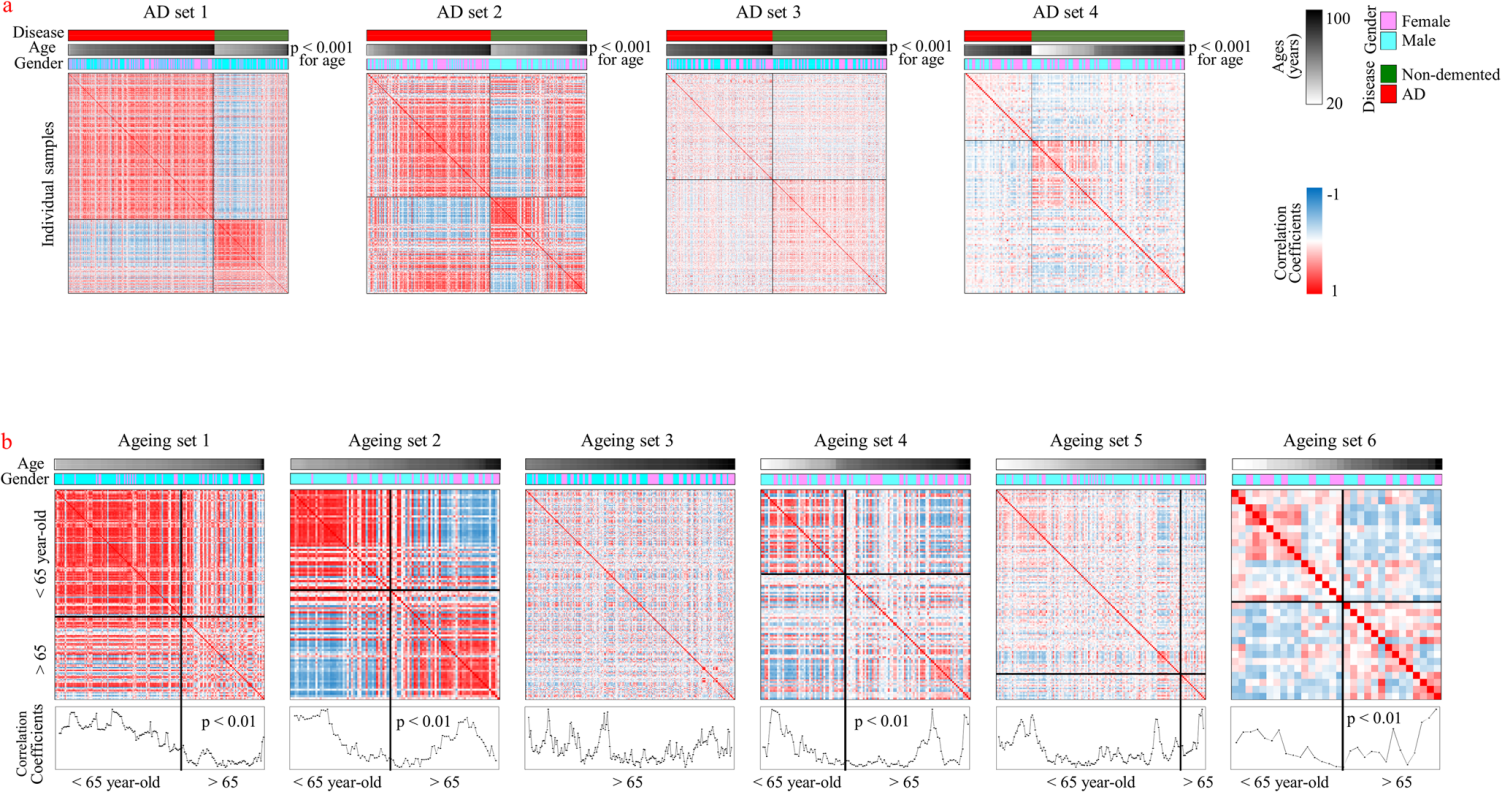
Correlation patterns of samples according to gene expression. Correlations were measured between individuals from **a** four AD sets and **b** six aging sets, using the log-based expression of genes. Information about samples and intensities of correlation (Pearson’s correlation coefficients) are shown in color bars. Samples were arranged according to age. **a** AD patient group was composed of more aged individuals than was the nondemented group in all AD sets (*p* < 0.001). **b** For aging sets, the levels of moving averaged coefficients of correlation are indicated. The age of 65 years was designated as the threshold line. Differences in the coefficients of samples from individuals who were older and younger than 65 years were measured using Student’s *t* test. Aging sets 1, 2, 4, and 6 showed significant differences in correlation (*p* < 0.01) between younger and older individuals

We identified genes associated with AD by analyzing microarray information archived in the GEO database. For this purpose, four AD datasets (AD sets 1–4) and six aging datasets (aging sets 1–6) comprising eight external microarray datasets (GSE15222, GSE44772, GSE33000, GSE48350, GSE5281, GSE53890, GSE30272, and GSE1572) [1, 35–41] were imported into our analysis platform. These public microarray datasets deal with diverse brain tissues from AD patients and/or nondemented individuals. For the present analysis, samples were limited to the cortex or hippocampus regions of the brain. AD sets were composed of AD cases and nondemented individuals, and aging sets were composed of only nondemented individuals from each microarray dataset. The characteristics of each microarray dataset are compared in Supplementary Table 1. The relationship between individuals was examined based on whole gene expression by measuring the correlation patterns among them. As shown in Fig. 2a, patients with AD and nondemented individuals showed different patterns of correlation. However, because the AD patient group was composed of more aged individuals than was the nondemented group in all AD sets (*p* < 0.001), it was necessary to adjust for the effects of age on gene expression. Therefore, we examined the effects of age by assessing the correlation patterns according to age among nondemented individuals (Fig. 2b). Interestingly, in many datasets (aging sets 1, 2, 4, and 6), younger individuals were correlated with younger individuals and older individuals were correlated with older individuals (*p* < 0.01). Although we did not determine the exact age threshold that discriminated the young from old groups in the present analysis, the age of 65 years, which is generally used to characterize late-onset AD, would be appropriate [42]. Thus, we excluded samples from individuals under the age of 65 from the present research of AD. The clustering patterns of whole gene expression among samples exclusively from individuals over 65 years of age are shown in Supplementary Fig. 1, in which we confirmed the segregation between AD cases and nondemented individuals and which indicates the presence of many differentially expressed genes in patients with AD.

### Selection of AD Genes

**Fig. 3:**
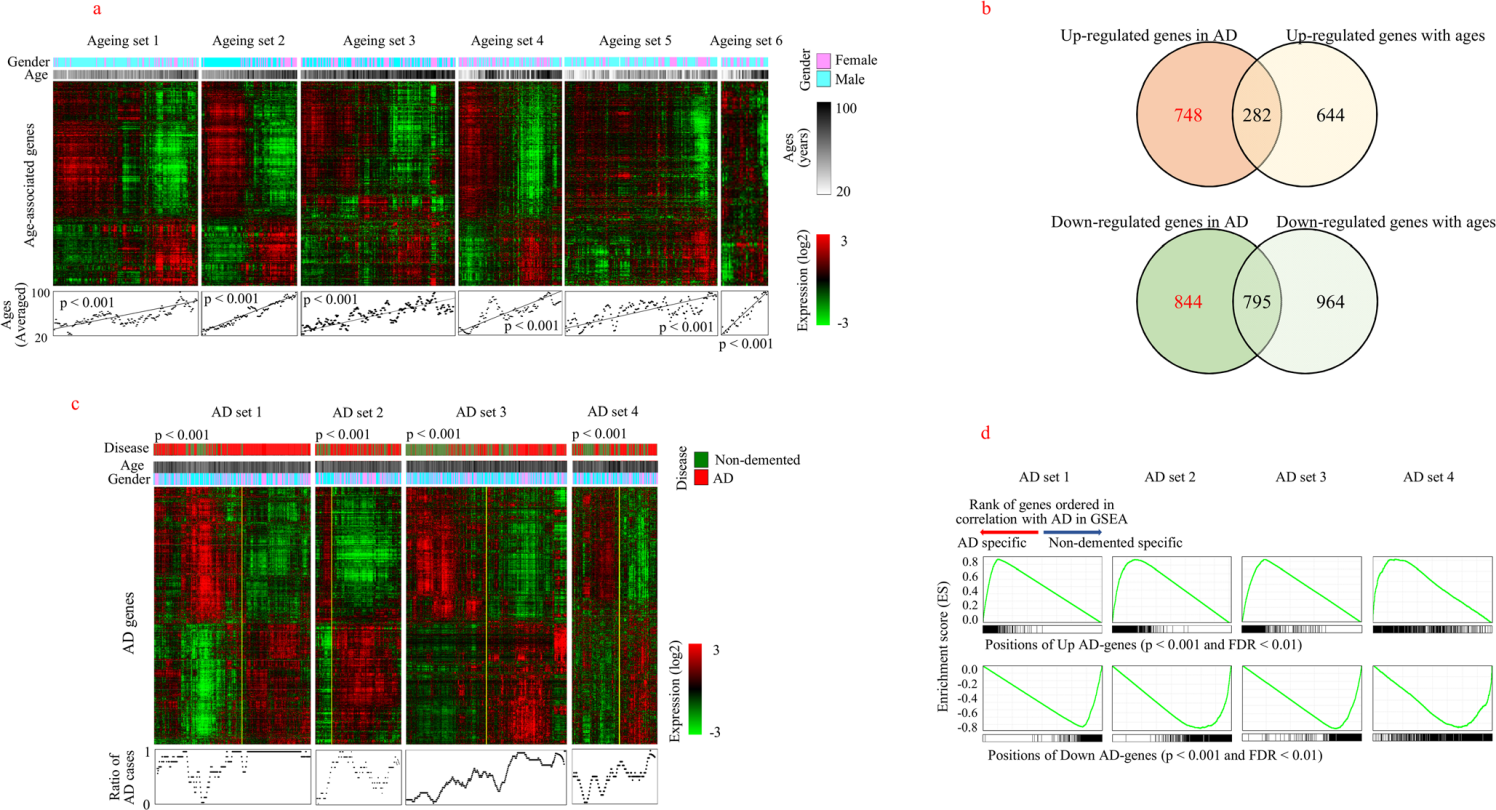
Selection of genes associated with AD. **a** Genes associated with aging (FDR < 0.01) were selected from SAM and hierarchically clustered. The distribution of the moving averaged age of samples is plotted in the lower region of the expression profile image with *p* value from general regression analysis. **b** The distribution of initial AD-associated genes and age-associated genes is shown in the Venn diagrams. Genes exclusively included in AD were finally selected as upregulated and downregulated AD genes. **c** Finally, selected AD genes were hierarchically clustered based on expression levels. Two subgroups of samples in each dataset were discriminated with yellow lines. Differential distribution of AD and nondemented individuals in the two subgroups (*p* < 0.001) was measured using chi-squared test with Yates’s continuity correction. The rate of AD samples in clustering profiles is also presented in the lower region of the image. **d** The effectiveness of AD genes in discriminating AD samples from nondemented samples was verified using GSEA. Upregulated and downregulated AD genes were enriched in the AD and nondemented groups, respectively, in each dataset (*p* < 0.001 and FDR < 0.01)

Although we excluded samples from individuals aged under 65 years, we further adjusted for age effects by eliminating age-associated genes. For this second-line adjustment for age effects, we selected age-associated genes from nondemented samples using the SAM algorithm under the option of quantitative response type [34]. The plot of gene distribution in SAM is shown in Supplementary Fig. 2. Age-associated genes were selected as commonly regulated genes (FDR < 0.01) in at least two datasets (Supplementary Fig. 3). Finally, we selected 761 and 1561 genes as being upregulated and downregulated age-associated genes, respectively. The validity of age-associated genes is shown in Fig. 3a, which indicates the presence of a close relationship between age and the expression levels of age-associated genes (*p* < 0.001).

To obtain a defined set of AD genes, we also implemented the SAM algorithm under the two-class discrimination option. The resulting differentially expressed genes (DEGs) between AD cases and nondemented individuals aged over 65 years were selected (FDR < 0.01) from four AD sets. For AD sets 1 and 2, the threshold value was set more stringently at FDR < 0.001, to reduce the number of selected genes. The distribution of genes and the threshold for the determination of gene selection in SAM are shown in Supplementary Fig. 4. To eliminate the bias that is inherent in each microarray dataset, we isolated genes that were commonly expressed in more than two datasets. The number of selected genes among datasets is shown in Supplementary Fig. 5. A total of 980 and 1550 genes were selected as the initial upregulated and downregulated AD genes, respectively.

The relationship between these initial AD genes and age-associated genes is shown in a Venn diagram, from which 282 (27.3% of the initial AD genes) upregulated and 795 (48.5% of the initial AD genes) downregulated genes were commonly selected (Fig. 3b). The significant overlap of genes observed supports the contention that the molecular features of AD are closely related with aging processes. Finally, by removing common genes from the initial AD genes, we selected 748 and 844 genes as final upregulated and downregulated AD genes, respectively. This final selection of genes was termed AD genes. The expression profile shows that AD genes discriminated AD cases from nondemented individuals (*p* < 0.001), as shown in Fig. 3c. AD genes are listed in Supplementary Table 2. The validity of AD genes was also confirmed by implementing a GSEA in each dataset. As shown in Fig. 3d, upregulated or downregulated AD genes were highly enriched in individuals with AD or nondemented individuals (*p* < 0.001 and FDR < 0.01), respectively, in all datasets, thus proving the specific expression of AD genes in the AD population.

### Functional Association of AD Genes

**Fig. 4:**
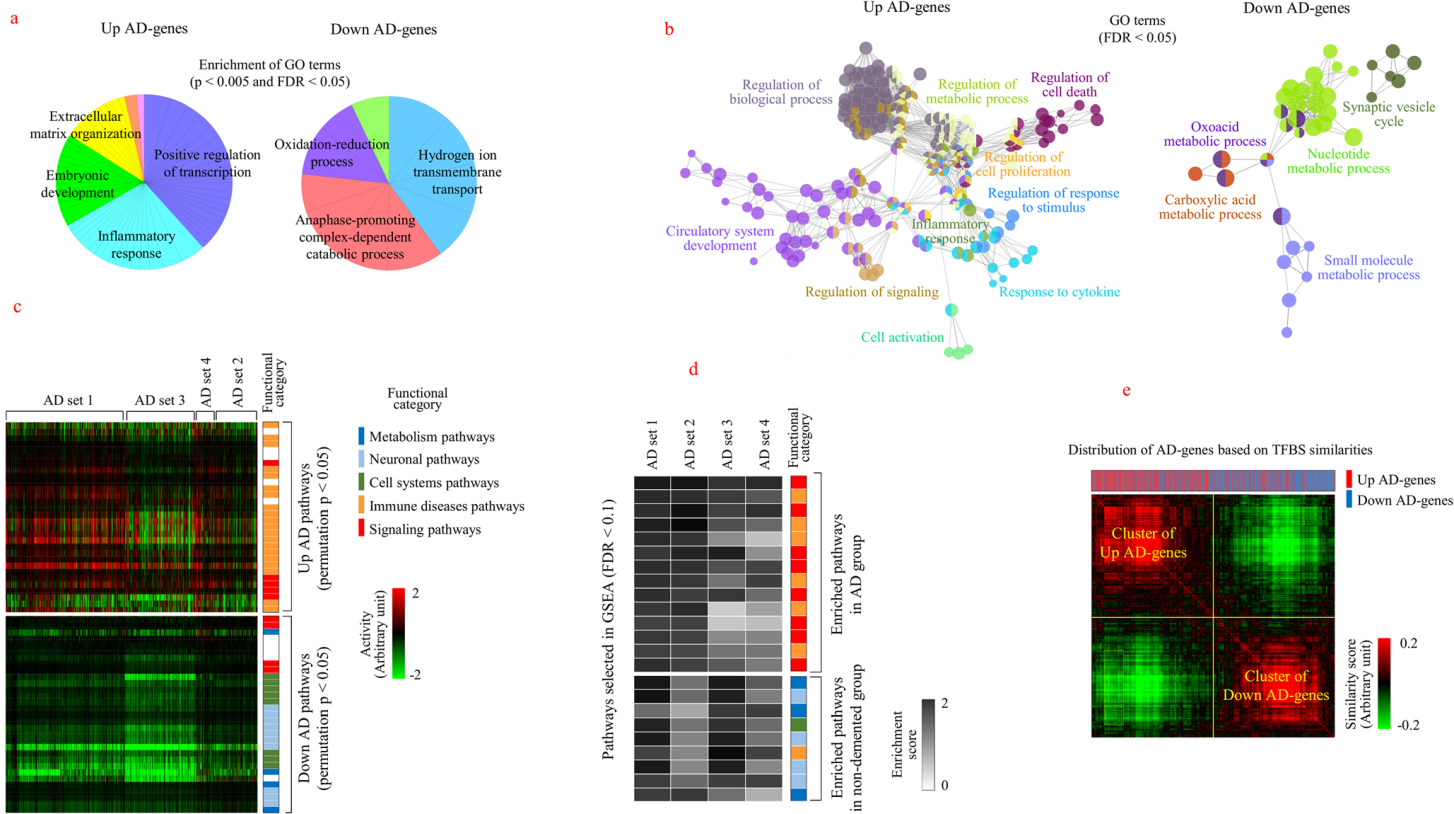
Functional characterization of AD genes. **a** The enrichment of GO terms associated with AD genes was measured using the DAVID and REVIGO program. Representative GO terms and their related nonredundant terms (*p* < 0.005 and FDR < 0.05) were presented in identical colors. **b** The functional connections of enriched GO terms associated with AD genes were measured using ClueGO. The related GO terms were segregated in each cluster (FDR < 0.01) and represented using the same color. The representative GO terms are indicated for each cluster. The size of node represents the significance of the GO term. Functionally related GO terms are partially overlapped. **c** Pathways that were differentially activated between AD and nondemented individuals (permutation *p* < 0.05) were selected, and their activities are visualized using a color scale. Columns represent individual samples, and rows represent pathways. The functional categories of pathways are displayed in color bars. **d** Pathways that were enriched commonly in all datasets (FDR < 0.1) were selected using GSEA. Enrichment scores for each pathway are represented as black and white intensities, as shown in the scale bar. **e** Upregulated and downregulated AD genes were clustered hierarchically based on the similarity of the TFBS structure in the promoter region (− 2000 to + 500 bp from the transcription start site). The positions of upregulated and downregulated AD genes are highlighted in the upper bar using red and blue colors, respectively. Differential distribution of AD genes between the two clusters was analyzed using chi-squared test with Yates’s continuity correction (*p* < 10^−5^). The color bar indicates the level of similarity, from red (i.e., high) to green (i.e., low), with arbitrary units

We then evaluated the functional implication of AD genes using a simple enrichment analysis of pathways and GO. Signaling-related functions, such as inflammatory response, cell cycle, and apoptosis, were associated with upregulated AD genes, whereas various metabolism-related functions, such as oxidative phosphorylation, ion transport, and amino acid metabolism, were associated with downregulated AD genes (*p* < 0.005 and FDR < 0.05), as shown in Table 1. In particular, pathways related to neurological diseases, including AD, Huntington’s disease, and Parkinson’s disease, were associated with downregulated AD genes. This functional segregation between upregulated and downregulated AD genes was more clearly demonstrated in the distribution of the nonredundant GO terms that were selected using the REVIGO algorithm [27], as shown in Fig. 4a. Signaling functions, such as transcriptional regulation, development, and inflammation, were enriched in upregulated AD genes, whereas metabolic functions, such as ion transport, catabolic processes, and oxidation–reduction, were enriched in downregulated AD genes. The functional segregation and differential expression of a large number of genes in AD cases imply the presence of a complex interaction of genes and functions during the processes leading to the pathological conditions of AD. Therefore, we examined the manner in which AD-associated functions are interconnected with each other using a network analysis. Various signaling functions associated with upregulated AD genes, including inflammatory response, cell death, and development, were grouped in a large cluster (FDR < 0.01). Metabolic functions associated with downregulated AD genes, which included nucleotide metabolism, carboxylic acid metabolism, and fatty acid metabolic processes, were also closely connected with each other (Fig. 4b). This interconnection of AD-associated functions may be the cause of the diverse pathological traits observed in AD. We also evaluated the altered activities of pathways (permutation *p* < 0.05) in AD cases and compared them with that observed for nondemented individuals using the expression levels of all genes included in each pathway, rather than using only AD genes. Immune-related pathways were predominantly activated, whereas neurological pathways and metabolic pathways were suppressed in AD individuals (Fig. 4c). This pattern of functional differentiation was confirmed in a GSEA, which showed that signaling pathways (including disease pathways and immune pathways) were enriched, whereas metabolic pathways and neurological pathways were depleted in the AD group (FDR < 0.1, Fig. 4d). A detailed list of these pathways is provided in Supplementary Fig. 6. A similar segregation of enriched GO terms was also observed using GSEA, as shown in Supplementary Fig. 7. As gene expression is determined by the type of transcription factor that binds to the promoter sequences, we analyzed the patterns of TFBS in AD genes. Figure 4e shows that AD genes were segregated into two major clusters based on TFBS similarity. Upregulated and downregulated AD genes were differentially distributed into each cluster, with statistical significance (*p* < 10^−5^), which indicated that the reciprocal expression of AD genes could be predetermined at the transcriptional level.

**Table 1:** GO terms and pathways enriched in AD genes

GO analysis							
GO ID	Up-AD genes	*p* value^a^	FDR^b^	GO ID	Down-AD genes	*p* value	FDR
0045944	Positive regulation of transcription	1.06E−09	3.47E−06	1902600	Hydrogen ion transmembrane transport	2.96E−04	3.06E−02
0006954	Inflammatory response	2.83E−09	4.63E−06	0031145	Anaphase-promoting complex-dependent catabolic process	6.41E−04	3.35E−02
0000122	Negative regulation of transcription	3.01E−08	3.29E−05	0051436	Negative regulation of ubiquitin-protein ligase	1.02E−03	4.56E−02
0006974	Cellular response to DNA damage stimulus	2.97E−07	2.43E−04	0016192	Vesicle-mediated transport	1.06E−03	4.69E−02
0043123	Positive regulation of I-κb kinase/NF-κb	5.23E−07	3.43E−04	0070125	Mitochondrial translational elongation	1.19E−03	4.34E−02
0001701	In utero embryonic development	1.89E−06	1.03E−03	0015031	Protein transport	1.31E−03	4.08E−02
0008285	Negative regulation of cell proliferation	4.74E−06	2.21E−03	0017156	Calcium ion regulated exocytosis	1.37E−03	4.74E−02
0006366	Transcription from RNA pol II promoter	1.08E−05	4.41E−03	0006123	Mitochondrial electron transport	1.43E−03	4.48E−02
0045893	Positive regulation of transcription	1.19E−05	4.33E−03	0051437	Positive regulation of ubiquitin-protein ligase	1.73E−03	4.69E−02
0071260	Cellular response to mechanical stimulus	2.38E−05	7.76E−03				
0010629	Negative regulation of gene expression	3.64E−05	1.07E−02				
0043066	Negative regulation of apoptotic process	4.19E−05	1.13E−02				
0030198	Extracellular matrix organization	4.37E−05	1.09E−02				
0042127	Regulation of cell proliferation	5.82E−05	1.35E−02				
0008283	Cell proliferation	8.35E−05	1.80E−02				
0008630	Intrinsic apoptotic signaling pathway	9.09E−05	1.84E−02				
0045931	Positive regulation of mitotic cell cycle	9.38E−05	1.79E−02				
0045669	Positive regulation of osteoblast differentiation	1.28E−04	2.29E−02				
0071480	Cellular response to gamma radiation	1.34E−04	2.29E−02				
0030199	Collagen fibril organization	1.35E−04	2.17E−02				
0001525	Angiogenesis	2.77E−04	4.22E−02				
0030308	Negative regulation of cell growth	3.64E−04	4.26E−02				
0050900	Leukocyte migration	3.96E−04	4.48E−02				
Pathway analysis							
KEGG ID	Up-AD genes	*p* value^a^	FDR^b^	KEGG ID	Down-AD genes	*p* value	FDR
04210	Apoptosis	6.45E−04	1.47E−02	01100	Metabolic pathways	1.28E−08	3.07E−06
05133	Pertussis	8.04E−04	3.90E−02	00190	Oxidative phosphorylation	4.00E−07	4.80E−05
05200	Pathways in cancer	1.04E−03	3.99E−02	05016	Huntington’s disease	1.49E−05	1.18E−03
05166	HTLV-I infection	1.17E−03	4.27E−02	05010	Alzheimer’s disease	1.84E−05	1.10E−03
05221	Acute myeloid leukemia	1.22E−03	4.49E−02	05012	Parkinson’s disease	6.08E−05	2.91E−03
				04260	Cardiac muscle contraction	1.14E−04	4.53E−03
				04721	Synaptic vesicle cycle	4.50E−04	1.53E−02
				00330	Arginine and proline metabolism	1.58E−03	4.63E−02

^a^*p* values were calculated using Fischer’s test

^b^FDR corrections were calculated using the Benjamini–Hochberg procedure

### Network Analysis of AD Genes

**Fig. 5:**
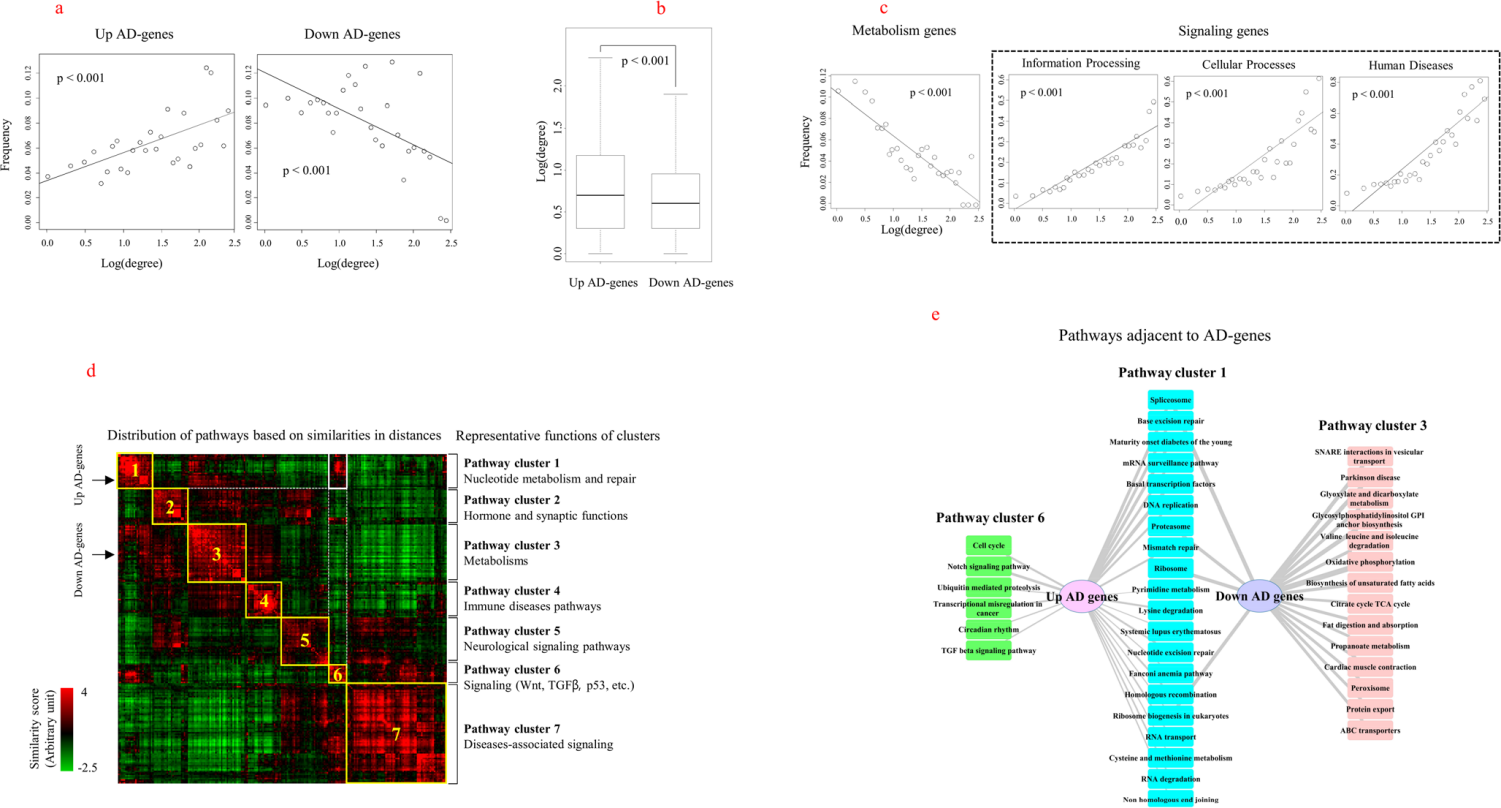
Network characteristics of AD genes. **a** The proportion of AD genes at each degree value was measured in a whole PPI network. Regression analysis was performed using generalized linear models. **b** The distribution of the degrees of node genes was compared between upregulated and downregulated AD genes. Statistical significance was assessed using Student’s *t* test. **c** The proportion of functionally related genes at each degree value was measured in a whole PPI network. Genes in each functional category were obtained from KEGG. **d** The distances of AD genes to pathways were examined by measuring the shortest path length among each gene set. The positions of upregulated and downregulated AD genes are indicated with arrows. Clusters of pathways were numbered according to representative biological functions, which are shown at the right side of the image. **e** Pathways that were positioned close to AD genes (similarity score > 3.35 corresponding to the highest 90%) were selected as AD-adjacent pathways. Edge thickness represents the level of the similarity score between two nodes. The colors of nodes indicate the biological functions of pathways, similar to the pathway clusters in the network

We further investigated whether this functional segregation of AD genes was also linked with positional segregation in the PPI network structure, as it was reported that the molecular function and position of genes are related in this network [43]. Before the characterization of AD genes in the network, we confirmed that both upregulated and downregulated AD genes exhibited the characteristics of a scale-free network (Supplementary Fig. 8). In a proportional plot of node degrees, we observed a reciprocal relationship between upregulated and downregulated AD genes. The proportion of genes with a high level of degrees was increased in upregulated AD genes, but was decreased in downregulated AD genes (Fig. 5a). In fact, the average of node degrees was higher in upregulated vs downregulated AD genes (*p* < 0.001) (Fig. 5b). We found that the general signaling and metabolic genes archived in KEGG also showed reciprocal patterns of node degree distribution, similar to that observed for upregulated and downregulated AD genes, respectively (Fig. 5c). This result confirmed that the functional segregation of AD genes, which was dependent on expression levels, may be correlated with positional segregation of genes in the PPI network. In addition to the degree distribution of genes, we measured the shortest path lengths between genes in the PPI network, to identify the nearest pathways to AD genes. First, we observed that near–distant gene sets were also functionally similar, based on genes in pathways. We then measured the pathways that included AD genes. As shown in Fig. 5d, pathways were grouped into seven clusters according to distance similarities. Each cluster of pathways represents a specific biological function. For example, pathway cluster 1, which included upregulated AD genes, is composed of nucleotide metabolism-related pathways, such as DNA repair and RNA transcription pathways. In addition, pathway cluster 1 was also correlated with signaling pathways, such as the Wnt, TGFβ, cell cycle, and p53 pathways of pathway cluster 6. Conversely, downregulated AD genes were closely correlated with metabolism-related pathways, such as glucose metabolism, amino acid metabolism, and fatty acid metabolism pathways in pathway cluster 3. During this process, we were not able to observe the size effects of pathways in measurements of distances between gene sets, as shown in Supplementary Fig. 9. The full list of pathways included in each cluster is shown in Supplementary Fig. 10. Pathways adjacent to AD genes with a similarity score > 3.35, corresponding to the top 90% pathways, were depicted in detail, as shown in Fig. 5e. It is evident that upregulated and downregulated AD genes were mainly linked with pathway clusters 1 and 3, respectively.

### Evaluation of Animal Models of AD

**Fig. 6:**
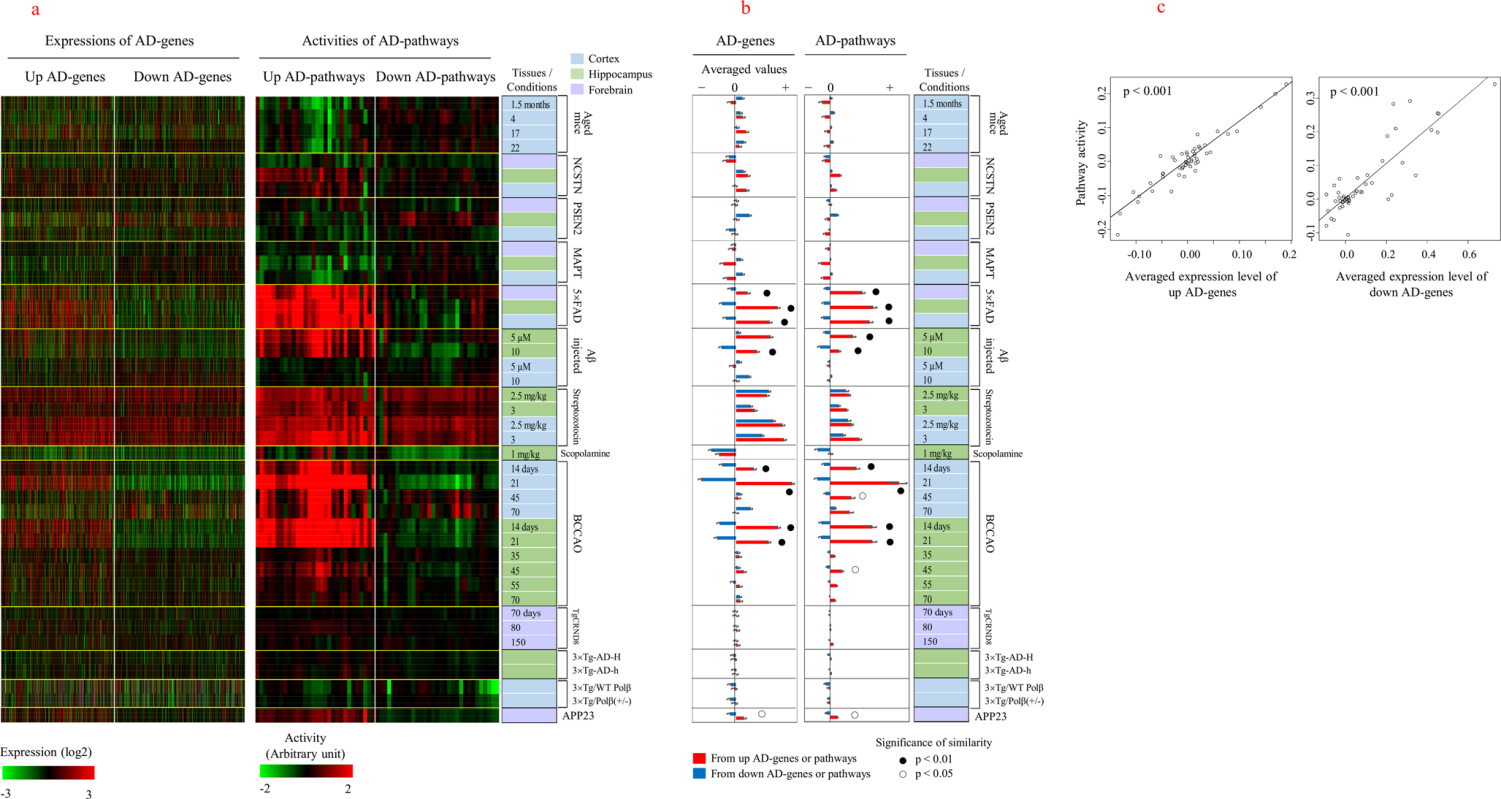
Functional evaluation of animal models. **a** Expression profile of AD genes and AD pathway activities in animal models. AD pathways were selected as those that were differentially activated between AD and nondemented individuals. **b** The expression levels of AD genes or AD pathway activities in animal models were quantitatively compared. Red and blue bars indicate averaged values obtained using upregulated AD genes (or repressed pathway activities) and downregulated AD genes (or depressed pathway activities), respectively. Animal models showing patterns of gene expressions that were similar to those of AD genes or activities of pathways similar to those of human AD are indicated in black (*p* < 0.01) and white (*p* < 0.05) circles and were determined based on a random permutation-based approach, as described in the “Materials and Methods” section. Values are presented as means ± standard errors. **c** The correlation was measured between the averaged expression levels of AD genes and pathway activities in animal models

**Fig. 7:**
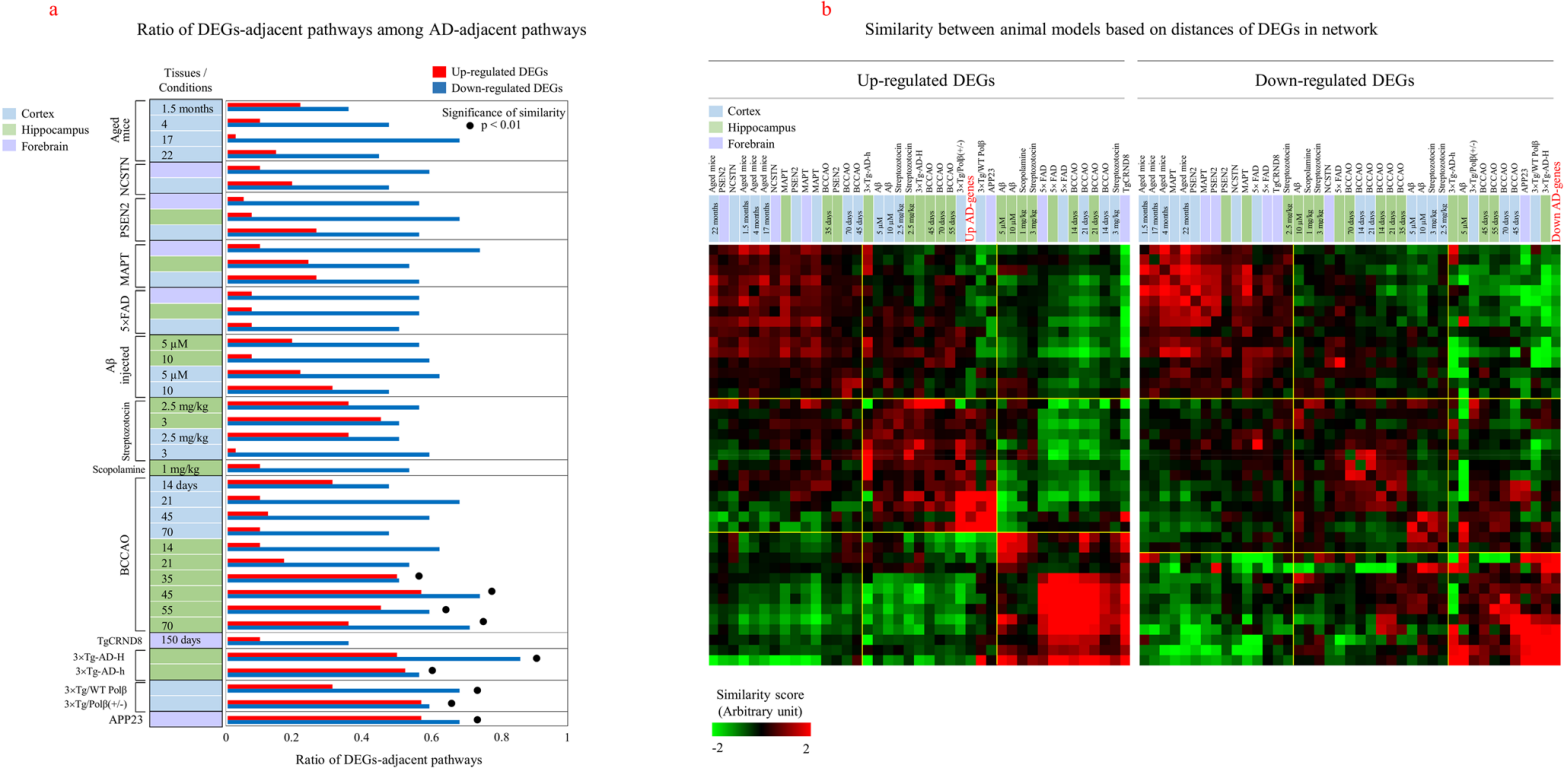
Network evaluation of animal models. **a** Distances between animal models and AD-adjacent pathways were examined by measuring the shortest path length using DEGs from animal models. AD-adjacent pathways were selected as the closest pathways to AD genes, as shown in Fig. 5e. Genes that were differentially expressed by at least 2-fold compared with normal controls in each animal model were selected as DEGs. For 3×Tg, APP23, and TgCRND8 models, genes with FDR < 0.01 were used as DEGs. The ratio of DEG-adjacent pathways (similarity score > 3.35) among AD-adjacent pathways was measured. Black circles indicate animal models showing a high similarity (*p* < 0.01) in pathway distribution to those observed for AD genes, as measured by random permutation-based calculation. **b** The similarities between animal models were measured based on distances of DEGs in the network. Animal models were then hierarchically clustered based on distances. The positions of upregulated and downregulated AD genes are highlighted in red color. Clusters of samples are indicated by yellow lines

The animal models used in the present study and their characteristics are listed in Table 2 and Supplementary Table 3. Among them, information pertaining to gene expression in TgCRND8 (GSE31372), 3×Tg-AD (GSE36981), 3×Tg/Polβ (GSE60911), and APP23 mice (GSE80465) was obtained from a public database. We compared the animal models regarding three aspects: (1) expression levels of AD genes, (2) activities of pathways, and (3) distance in the network. First, the expression levels of AD genes were compared among animal models. We found that 5×FAD mice (forebrain, hippocampus, and frontal cortex), the frontal cortex and hippocampus of rats in the initial phases (14 and 21 days) after BCCAO, and the hippocampus of Aβ-injected mice (10 μM) showed a similar pattern of gene expression compared with those of human AD genes (Fig. 6a). Intriguingly, we observed different patterns of gene expression and pathway activity depending on the regions (cortex and hippocampus) of the brain, particularly in Aβ-injected mice and BCCAO rat models (Fig. 6a). A quantitative plot of gene expression clearly showed the reciprocal regulation of AD genes in the three models mentioned above compared with the other models (Fig. 6b). In addition to these three models, APP23 animals also exhibited similar patterns of gene expression, although the expression levels were low. Similar clustering patterns of animal models with age-associated genes were observed, as shown in Supplementary Fig. 11, suggesting that these three models may also be used as models of aging. The activities of pathways that showed differential regulation in AD were measured in animal models (Fig. 6a). Consistent with the gene expression measurements, the pathway activities in 5×FAD mice, rats with BCCAO (initial phases), and Aβ-injected mice (hippocampus) were similar to those observed in human AD, as immune disease-related pathways were upregulated and metabolism and neurological pathways were downregulated (Fig. 6b). In fact, the averaged expression levels of AD genes were correlated with pathway activities among animal models (Fig. 6c). The two methods mentioned above were based on the direct use of the expression levels of genes. We then examined the distances of genes in the PPI network using DEGs rather than AD genes. DEGs were selected using 2-fold criteria for the animal models analyzed here, whereas FDR < 0.1 under the two-class response type option of SAM was used as a criterion for the selection of DEGs from animal models from the GEO database (TgCRND8, 3×Tg, and APP23 models). The plot of gene distribution from animal models in SAM is shown in Supplementary Fig. 12. Using DEGs, we determined the number of animal models that were connected to preselected pathways adjacent to AD genes, which were the nearest pathways from AD genes (Fig. 5e). We achieved this by measuring the distances between each animal model and AD-adjacent pathways. As shown in Fig. 7a, 3×Tg, APP23, and late-phase (45, 55, and 70 days after surgery) BCCAO models were positioned close to AD-adjacent pathways consisting of mainly pathway cluster 1 (nucleotide metabolism and repair pathways) and pathway cluster 3 (general metabolism pathways) for upregulated and downregulated AD genes, respectively (Fig. 5e). Subsequently, we compared directly the similarities in distances in the PPI network between animal models. For upregulated DEGs, 5×FAD mice, initial-phase (14 and 21 days after surgery) BCCAO rats, and Aβ-injected mice (hippocampus) were clustered in one group, whereas 3×Tg, APP23, and late-phase BCCAO models were clustered with upregulated AD genes in another group (Fig. 7b). The 3×Tg, APP23, and late-phase BCCAO models were also co-clustered with downregulated AD genes. The similarities of distances in the network observed between models suggest that the features of the 3×Tg, APP23, and late-phase BCCAO models are close to those of human AD. However, this result of distances in the network seems to be different from previous results of expression patterns and pathway activities, which showed that 5×FAD, initial-phase BCCAO, and Aβ-injected models (hippocampus) were more similar with human AD than were the other models. DEGs from each animal model were used to measure the distances in the network, rather than AD genes. Considering that there was a lack of overlap between AD genes and DEGs (Supplementary Fig. 13), distance in the network based on DEGs should be considered to provide results that are independent from those based on the direct comparison of AD gene expression. DEGs represent the most-altered genes in each animal model; therefore, they determine the phenotypic characteristics of each animal model, including AD-like pathological traits. Therefore, a closeness in distance between DEGs and AD genes implies that this model may be representative of the pathological characteristics of human AD. We summarized the results of these comparisons between animal models in Table 2.

**Table 2:** Functional evaluation of animal models

Animal models			Experiments^a^		
			Expressions of AD genes	Pathway activities	Distances in network to AD genes
Models	Conditions	Tissues			
Aged mice	1.5 months	Cortex			
	4 months				
	17 months				
	22 months				
NCSTN model		Basal forebrain			
		Hippocampus			
		Cortex			
PSEN2 model		Basal forebrain			
		Hippocampus			
		Cortex			
MAPT model		Basal forebrain			
		Hippocampus			
		Cortex			
5×FAD		Basal forebrain	●	●	
		Hippocampus	●	●	
		Cortex	●	●	
Aβ-injected model	5 μM	Cortex			
		Hippocampus		●	
	10 μM	Cortex			
		Hippocampus	●	●	
Streptozotocin model	2.5 mg/kg	Cortex			
		Hippocampus			
	3 mg/kg	Cortex			
		Hippocampus			
Scopolamine model	1 mg/kg	Hippocampus			
BCCAO	14 days after surgery	Cortex	●	●	
	21 days		●	●	
	45 days			O	
	70 days				
	14 days	Hippocampus	●	●	
	21 days		●	●	
	35 days				●
	45 days			O	●
	55 days				●
	70 days				●
TgCRND8 mice		Forebrain			
3×Tg-AD-H mice	Homozygous	Hippocampus			●
3×Tg-AD-h mice	Hemizygous	Hippocampus			●
3×Tg/WT Polβ		Cortex			●
3×Tg/Polβ(+/−)		Cortex			●
APP23 mice		Forebrain	O	O	●

^a^Black and empty circles indicate strong (*p* < 0.01) and weak (*p* < 0.05) relationships of models, respectively, at each experimental condition

## Discussion

Genetic studies have shown that early onset AD is caused by dominantly inherited mutations in *APP*, *MAPT*, *MPSEN1*, and *PSEN2*. The functional implications of these risk genes strongly suggest the amyloid cascade hypothesis, which postulates that changes in Aβ homeostasis lead to the aggregation and deposition of Aβ and, eventually, to the pathological conditions of AD. Interestingly, it has been reported that these genes can also be associated with late-onset AD, as rare variants [44–47]. However, a GWAS analysis showed that various biological functions that are not directly associated with Aβ physiology, including lipid (cholesterol) metabolism, immune response, and endocytosis, were involved in late-onset AD [48]. This molecular information regarding AD suggests that various biological functions are implicated in the pathophysiology of AD.

In the present study, many genes were also shown to be differentially expressed in AD cases compared with nondemented individuals, supporting the alteration of diverse biological functions in AD. The measurement of pathway activities showed that neurological pathways (neurological diseases, addiction-related pathways, and synaptic functions) and metabolic pathways (oxidative phosphorylation, amino acid metabolism, and the insulin pathway) were downregulated, whereas immune disease-related pathways were markedly upregulated in AD. In particular, immune functions have been reported as being among the primary targets of anti-AD drugs, as the activation of immune cells (microglia and astrocytes) and their related signaling pathways seems to be involved in neuroinflammation in AD [1, 49]. Therefore, the activation of pathways related to neuroinflammation and neuronal cell death observed in the present study seems to support the importance of the immune reaction in the process of AD. Conversely, the suppression of metabolism-related pathways and neurological disease pathways evidenced the presence of other important factors in the pathology of AD. Moreover, we observed a close proximity between metabolic pathways and neurological disease pathways in the network distance analysis of pathways. Many reports also indicated the presence of metabolic dysregulation in AD, such as dysregulation of the insulin pathway and the resulting impairment of glucose metabolism [50–52]. Consistently, in the present analysis, the insulin and energy metabolism pathways (such as oxidative phosphorylation) were suppressed or associated with downregulated AD genes. In addition, previous reports of an association between immunity and metabolic diseases, such as diabetes [53], suggest that whole biological functions that were shown to be enriched in the present study, such as immune function, metabolic functions, and neuronal disease pathways, could be interconnected to result in the pathological conditions of AD. Therefore, we speculate that, for the development of effective therapeutic agents against AD, multiple targets, including immune and metabolic functions, should be considered simultaneously.

Moreover, we observed that the topological segregation of AD genes was dependent on their expression levels. Upregulated genes, such as signaling-related genes, tended to be located more centrally in the PPI network, whereas downregulated genes, such as metabolism-related genes, tended to be located more peripherally in the network. In addition to the enrichment analysis of functions, a distribution analysis of AD genes in the PPI network confirmed the reciprocal segregation of the biological functions of AD genes and a close association between the function and topology of genes in the network. In accordance with the results of the direct measurement of pathway activity, upregulated and downregulated AD genes showed reciprocal associations with signaling pathways (pathway clusters 1 and 6) and metabolism pathways (pathway cluster 3), respectively. We reported previously the reciprocal regulation of biological functions between signaling pathways and metabolic pathways in diverse biological situations [15, 17, 18] which suggests that the reciprocal regulation of gene expression and biological functions may be the global regulatory mechanism that is predetermined in the promoter sequences of genes. Protein modification such as glycosylation, acetylation, and phosphorylation is also thought to play an important role in the reciprocal regulation of metabolism and signaling processes [54].

Based on the molecular features of AD described above, we assessed the similarity between the features of various animal models and those of human AD. Models of AD can be classified into two categories: genetically modified and nongenetically modified models. Regarding the former, we used transgenic models such as the 5×FAD, mutated PSEN, or wild-type MAPT overexpression models. In addition, we incorporated public sources of microarray datasets of widely used transgenic mice, such as the TgCRND8, APP23, and 3×Tg models. All transgenic models used here involved the overexpression of mutated genes associated with Aβ and/or tau pathophysiology, including *APP*, *NCSTN*, *PSEN1*, *PSEN2*, and *MAPT*. As these transgenic models are based on genes that are mutated in familial AD, they do not represent the molecular features of human sporadic late-onset AD. Therefore, as nongenetic models of AD or dementia, we incorporated various types of models, such as aged mice, models with BCCAO, i.c.v.-injected Aβ mice, i.c.v.-administered streptozotocin mice, and scopolamine-treated mice [10, 55]. In particular, rats with BCCAO, which have been used as models of vascular dementia, were induced by permanent occlusion of the bilateral common carotid arteries, to result in chronic cerebral hypoperfusion. The biphasic regulation of gene expression and associated functions in the BCCAO model were previously reported by us. In this model, immune functions were activated in the initial phase (14 and 21 days) after operation, while neurological functions were suppressed in later phases [15].

The measurement of the expression levels of AD genes revealed that three models, i.e., 5×FAD mice, the hippocampus of Aβ-injected mice, and initial-phase BCCAO rats, showed patterns that were consistent with those of human AD. A pathway activity analysis also demonstrated that three models were most similar with human AD, in which neurological and metabolic pathways were downregulated, while diverse immune disease pathways were markedly upregulated. Although we measured three animal models that mimic human AD, it was difficult to identify common molecular elements that were present in all three models, as the BCCAO model, unlike the other two models, is not a transgenic model aimed at interfering with Aβ physiology. However, the report that β-secretase 1 (BACE1) and Aβ were upregulated in BCCAO rats [56] may imply the importance of Aβ pathology in this model. Nevertheless, considering that the other transgenic models evaluated in the present study also exhibit direct or indirect dysregulation of Aβ physiology, our results suggest that there may be functional differences among Aβ-based animal models.

In addition to differences between models, we observed differences in expression patterns of AD genes depending on regions of the brain. In Aβ-injected mice and BCCAO rats especially, the cortex region showed different expression patterns of AD genes or age-associated genes when compared with those of the hippocampus. We speculated that this regional specificity could be attributed to the way each model was constructed. For example, Aβ delicately injected into the cerebral ventricle could affect the hippocampus and cortex regions in temporal sequence. In addition, occlusion of the bilateral common carotid arteries could result in selective damage to brain regions, although more supportive evidence should be provided. We expect that regional differences in the brain between animal models could provide important information about changes in cognitive function in AD, because each region in the brain has different roles in cognitive function and further provides different causal factors for cognitive decline in pathological conditions of AD [57, 58].

Conversely, the measurement of distances in the PPI network showed that late-phase (45, 55, and 70 days after surgery) BCCAO rats and transgenic mice such as the 3×Tg and APP23 models were positioned most closely to AD genes. Because we used DEGs from each animal model in this network analysis, rather than AD genes, the results pertaining to network distances should be considered separately from the results of AD gene expression. Therefore, it can be concluded that three models, 5×FAD mice, Aβ-injected mice, and initial-phase BCCAO rats, may be used as suitable models for the observation of the pathway activities and expression levels of AD genes that characterize the molecular features of human AD, whereas late-phase BCCAO and transgenic models (including 3×Tg and APP23 models) may be used for the observation of phenotypic changes resulting from model-specific genes as molecular targets for the evaluation of novel drugs.

In this research, we included only individuals who were aged over 65 years for the isolation of genes associated with late-onset AD. However, taking into consideration that aging is a critical factor in late-onset AD, age-related changes in gene expression may affect greatly the processes of onset and further progression of AD. As shown in Fig. 2b, the pattern of overall gene expression was different between young (under age 65) and old (over age 65) individuals, evidencing changes in the expression of numerous genes during the process of aging, as reported previously at the genome level [59]. The close relationship between AD genes and age-associated genes can also be verified by the close distances of these two sets of genes in the PPI network, as shown in Supplementary Fig. 14. This similarity may provide an explanation for the high prevalence of AD among the elderly population, although the factors that trigger the onset of AD remain to be identified. Although we mainly focused on AD rather than the aging process in the present study, considering that age is a definite risk factor for late-onset AD, the causal effects of age in development of AD should be investigated. The recent view that AD is initiated decades before clinical manifestation of cognitive decline suggests that AD could be on a continuum of aging in the brain [60, 61]. Animal models, especially mice, have been reported to show age-related losses of cognitive function [62, 63], which suggests that aged-animal models could be applied to AD research related to cognitive aging. However, in the brain of aged mice, we did not observe any significant changes in the expression of AD genes or age-associated genes. A previous report also showed that only a small number of genes have evolved to be coexpressed in the brain in humans and mice [59], suggesting differences in their aging process. Therefore, aged mouse models should be used carefully in research of aging or aging-related diseases.

In conclusion, we identified a group of AD genes from multiple sources of gene expression datasets and observed their reciprocal regulation with specific biological functions, which were dependent on expression levels. Our results may explain the diverse pathological aspects of AD, such as the immunological activation and impaired glucose metabolism that are observed often in patients with AD. Based on these molecular features, we assessed the similarities between AD animal models and human AD. The 5×FAD, Aβ-injected mouse, and initial-phase BCCAO rat models showed patterns of gene expression that were similar to those of AD genes. However, when we used DEGs from each animal model in a topological measurement of distances, 3×Tg, APP23, and late-phase BCCAO models were positioned close to AD genes. Therefore, in the development of therapeutic agents against AD, multitarget approaches affecting multiple functions, such as signaling, immune, and metabolic functions, should be considered first and appropriate animal models should be used, depending on the specific targets that are to be evaluated in the measurement of drug effectiveness at the molecular level.

## Electronic Supplementary Material

ESM 1 (DOCX 21409 kb)
