# Supplementary material for: Evaluation of Animal Models by Comparison with Human Late-Onset Alzheimer’s Disease
Source: Mol Neurobiol. 2018 Apr 14;55(12):9234–50. doi: 10.1007/s12035-018-1036-6 (PMC6208860; doi:10.1007/s12035-018-1036-6)
Supplement: Supplementary file 1 — (DOCX 21409 kb) [file 12035_2018_1036_MOESM1_ESM.docx]

**[Supplementary materials]**

**Evaluation of animal models by comparison with human late-onset Alzheimer’s disease**

Bu-Yeo Kim^1,†,^**^*^**, Hye-Sun Lim^1,†^, Yoonju Kim^1^, Yu Jin Kim^1,2^, Imhoi Koo^3^, Soo-Jin Jeong^1,4^

^1^ Herbal Medicine Research Division, Korea Institute of Oriental Medicine, Daejeon, Republic of Korea

^2^ College of Pharmacy, Chungnam National University, Daejeon, Republic of Korea

^3^ Huck Institutes of Life Sciences, Pennsylvania State University, PA, United States of America

^4^ Korean Medicine of Life Science, University of Science & Technology, Daejeon, Republic of Korea

**Supplementary Table 1. Characteristics of the public datasets used in the present study**

| AD  Experiment | Ageing  Experiment | Individual  GEO ID | Brain regions | Sample size | | Reference |
| --- | --- | --- | --- | --- | --- | --- |
|  |  |  |  | AD | Non-demented |  |
| AD set 1 | Ageing set 1 | GSE33000 | Cortex | 310 | 156 | [17] |
| AD set 2 | Ageing set 2 | GSE44772 | Cortex | 129 | 100 | [22] |
| AD set 3 | Ageing set 3 | GSE15222 | Cortex | 174 | 187 | [21] |
| AD set 4 | Ageing set 4 | GSE48350 | Cortex and hippocampus | 80 | 173 | [2] |
|  |  | GSE5281 | Cortex and hippocampus | 87 | 74 | [14] |
|  |  | GSE53890 | Cortex | - | 41 | [16] |
|  | Ageing set 5 | GSE30272 | Cortex | - | 148 | [5] |
|  | Ageing set 6 | GSE1572 | Cortex | - | 30 | [15] |

**Supplementary Table 2. Full list of AD-genes**

| UP-regulated AD-genes | | | | | | | | |  | DOWN-regulated AD-genes | | | | | | | | | |
| --- | --- | --- | --- | --- | --- | --- | --- | --- | --- | --- | --- | --- | --- | --- | --- | --- | --- | --- | --- |
| AAMDC | CCDC102A | DOCK6 | GPSM3 | LILRA5 | NINJ1 | RASL12 | SLC7A7 | TNIP1 |  | ABCA5 | BSCL2 | CSTF2T | EXOSC9 | HSD17B8 | MCCC2 | NTMT1 | PSMD12 | SNX10 | TRAPPC13 |
| AATF | CCDC117 | DOK1 | GRAMD3 | LILRB3 | NKX3-1 | RASSF1 | SLC7A9 | TNPO1 |  | ABCE1 | C11orf57 | CTC1 | EXTL1 | HSPB11 | MCEE | NTPCR | PSMD14 | SNX24 | TRAPPC2L |
| ABCA1 | CCDC120 | DPP9 | GRN | LIMK2 | NPFF | RBBP6 | SLPI | TNXB |  | ABR | C12orf10 | CTH | F8 | HSPB3 | MCM4 | NUAK1 | PSMG3 | SNX4 | TRAPPC6A |
| ABCA7 | CCDC149 | DRAM1 | GSDMD | LIN9 | NPNT | RBCK1 | SMAD1 | TP53INP1 |  | ACAD9 | C14orf2 | CTNNBIP1 | FABP6 | HSPBP1 | ME2 | NUDT11 | PTGFRN | SNX8 | TRHDE |
| ABCB7 | CCDC88B | DSE | GTF3C5 | LINC00260 | NPRL3 | RBM14 | SMAD4 | TPD52L1 |  | ACBD6 | C14orf79 | CTSV | FAM127A | IFT27 | MEAF6 | NUDT18 | PTH2R | SORCS3 | TRIM37 |
| ABHD4 | CCDC9 | DTX2 | GYG2 | LINC00310 | NRIP2 | RBM15B | SMAD5 | TPM2 |  | ACLY | C16orf59 | CUL3 | FAM133A | IFT52 | MECR | NUDT2 | PYCR2 | SPAG7 | TRIM45 |
| ABL1 | CCL2 | DTX3L | HAP1 | LMAN2L | NT5DC2 | RBM4 | SMARCC1 | TPRA1 |  | ACOT4 | C17orf58 | CWF19L1 | FAM13C | IGFBPL1 | MEMO1 | NUDT6 | PYCRL | SPATA7 | TRIM48 |
| ABTB2 | CCNT1 | DTYMK | HAVCR2 | LMNA | NXN | RBM47 | SMG5 | TREM2 |  | ACRV1 | C17orf75 | CYB561D2 | FAM160A2 | IMMP1L | MESP1 | NUPL2 | QPCT | SPATS2L | TRIM54 |
| ACACB | CCR1 | DUSP1 | HDAC4 | LOC142937 | NXPE2 | RBM48 | SMG6 | TRIM38 |  | ACSL4 | C18orf21 | CYP2C8 | FAM163A | IMMP2L | METTL22 | NYAP1 | R3HDM2 | SPCS1 | TRIM9 |
| ACAN | CCRL2 | EDC3 | HDAC7 | LOC439911 | NXT1 | RBM6 | SMO | TRIM8 |  | ACSL6 | C19orf48 | CYP2E1 | FAM213B | IMMT | MIEF2 | OAT | RAB39B | SPHKAP | TRMT10C |
| ACKR1 | CD151 | EFCAB14 | HDGF | LOXL2 | OGFRL1 | RCC1 | SMOX | TRIP10 |  | ACTG1 | C1orf61 | CYP4X1 | FAM216A | IMP3 | MKRN2 | OCA2 | RAB3B | SPINT2 | TRMT61B |
| ACSF2 | CD34 | EFNA1 | HEXDC | LPIN1 | OGN | REEP4 | SMTN | TROVE2 |  | ACTN2 | C1QTNF9 | CYTH2 | FAM3C | IMP4 | MKX | OCIAD1 | RAB40B | SPON2 | TRPC1 |
| ACSL1 | CD59 | EGLN2 | HIGD1B | LRP10 | OLAH | RELA | SND1-IT1 | TTC38 |  | ACTR6 | C2orf27A | DCHS2 | FAM81A | INA | MNAT1 | OCM2 | RAB6C | SPRED1 | TRUB1 |
| ACVR2B | CD93 | EIF4EBP1 | HIST1H1C | LRR1 | OR2H2 | RERE | SNX33 | TUBB6 |  | ADCK1 | C3orf17 | DCLK1 | FAM83H | INHA | MOCS2 | OGFOD3 | RABEPK | SPRED2 | TSPAN13 |
| ADORA2A | CDC25B | ELF1 | HIST1H2BD | LRRC37A | ORAI3 | REXO4 | SORBS1 | TUBD1 |  | ADCYAP1 | C4orf27 | DCTN6 | FANCF | INSM2 | MORF4L2 | OPALIN | RALYL | SPRY4 | TSPAN7 |
| AGFG2 | CDC42EP4 | ELK1 | HIST2H2BE | LSMEM1 | OSM | RFTN2 | SOWAHC | UBALD2 |  | ADK | C7orf50 | DDA1 | FASTKD3 | INTU | MPI | OPN3 | RANBP1 | SPRYD3 | TSPYL4 |
| AGTRAP | CDH23 | EML3 | HLX | LTBP2 | P2RX7 | RFX2 | SOX12 | UBTD1 |  | ADO | C9orf24 | DDX24 | FBXL14 | IPCEF1 | MPL | ORC3 | RAP1GAP | SPRYD7 | TSR2 |
| AIFM3 | CDK13 | EMX2 | HMHA1 | LTBR | P4HA2 | RFX4 | SOX9 | UCK1 |  | AGAP2 | C9orf91 | DDX28 | FBXL16 | ISCU | MPO | OSBPL10 | RAPGEF4 | SPTSSB | TSTA3 |
| AIM2 | CDK2AP1 | ENGASE | HMOX1 | LYAR | PACSIN2 | RGCC | SPAG9 | UIMC1 |  | AHI1 | CACNA2D3 | DET1 | FBXL2 | ITIH4 | MPP1 | OSBPL1A | RAPGEFL1 | SRM | TTC3 |
| AJUBA | CDK2AP2 | ENTHD2 | HNRNPUL1 | LYL1 | PAFAH1B3 | RGL2 | SPARC | USF2 |  | AIG1 | CACNB1 | DFNA5 | FBXL22 | ITPA | MPV17 | OVOS2 | RBFOX1 | SRP19 | TTC30A |
| AK4 | CDKN1A | EP300 | HOMER3 | LYN | PALD1 | RGS3 | SPHK1 | USP3 |  | AIMP2 | CALM2 | DGUOK | FBXO27 | JADE1 | MRAP2 | P2RX2 | RBFOX2 | SRSF3 | TTPAL |
| AKAP8L | CEBPB | EPB41L5 | HP | LZTR1 | PAPD5 | RHBDF1 | SPR | UXT |  | AK5 | CAMLG | DHDDS | FBXO3 | JAKMIP1 | MROH7 | PACSIN1 | RBM45 | SRSF7 | TTYH1 |
| AKR1C1 | CEP135 | EPHA2 | HP1BP3 | MAF1 | PARVG | RHOC | SQRDL | VAMP8 |  | AKAP6 | CARTPT | DHRS11 | FBXO9 | KALRN | MRPL16 | PAM | RBMX | SRSF8 | TUB |
| AKR1C2 | CEP350 | ERAP2 | HRASLS2 | MAFF | PAX6 | RHOJ | SQSTM1 | VAT1 |  | ALDH5A1 | CBLN2 | DIRAS1 | FECH | KCNB2 | MRPL2 | PAQR9 | REPS1 | SSSCA1 | TUBA1B |
| AKR1C3 | CFD | ESAM | HSD11B2 | MAML1 | PBX3 | RIC8B | SRGAP1 | VEGFB |  | ALDOA | CCDC103 | DIRAS2 | FGGY | KCNIP4 | MRPL20 | PARP2 | RERG | STAMBPL1 | TUBA1C |
| AMICA1 | CFLAR | EVA1B | HSD17B13 | MAP3K3 | PCED1A | RIN2 | SRRT | VEZF1 |  | ALG14 | CCDC106 | DIRAS3 | FH | KCNJ4 | MRPL24 | PCCB | RHEB | STAR | TUBG2 |
| ANAPC16 | CGNL1 | EYA2 | HSD17B7 | MAP3K5 | PCF11 | RNASE3 | SRSF4 | VMO1 |  | ALKBH6 | CCDC113 | DIRC2 | FIG4 | KCNN2 | MRPL32 | PCSK1 | RHOBTB2 | STARD3NL | TYRP1 |
| ANG | CHD1 | F11R | HSD3B7 | MAPK4 | PCOLCE | RNF152 | ST5 | VNN2 |  | ALOX12B | CCDC115 | DLEU7 | FKBP3 | KCNQ3 | MRPL40 | PCSK2 | RIIAD1 | STAT4 | U2AF1L4 |
| ANKFY1 | CHDH | FAM129B | HSF1 | MAPKAPK2 | PDGFRB | RNPEPL1 | ST6GALNAC2 | VTI1A |  | AMDHD2 | CCDC53 | DLGAP2 | FN3KRP | KCTD13 | MRPL54 | PCYOX1L | RIMKLA | STK39 | UBC |
| ANKRD35 | CHST12 | FAM167B | HSPB2 | MBD3 | PDIA4 | RP2 | STAB1 | WASH2P |  | AMIGO2 | CCDC65 | DMKN | FRMD4A | KHDRBS1 | MRPS18A | PDCD2L | RIMS2 | STMN3 | UBE2V1 |
| ANTXR2 | CIC | FAM181A | HVCN1 | MCL1 | PDPN | RPN2 | STAG2 | WDFY2 |  | ANAPC10 | CCDC96 | DMRTC1 | FUNDC1 | KIAA0319 | MRPS22 | PDE6D | RIMS3 | STX12 | UBLCP1 |
| ANXA2 | CLCN7 | FAM189A2 | HYAL1 | MCM3 | PDZK1 | RPS6KA5 | STARD10 | WDR6 |  | ANAPC15 | CCIN | DNAJC27 | FXYD6 | KIAA1217 | MRPS25 | PDHA1 | RMI2 | STXBP1 | UBQLN2 |
| ANXA5 | CLDN5 | FAM43A | HYAL2 | MCM5 | PFKFB3 | RPS6KB1 | STARD3 | WDR81 |  | ANKRD13C | CCNB1 | DNAL4 | GABBR1 | KIAA1324 | MRPS35 | PDHB | RNASEH2B | SULT4A1 | UBXN8 |
| APPL2 | CLEC18A | FAM63A | HYI | MECOM | PGAM2 | RRP1 | STARD7 | WNK1 |  | ANKRD29 | CCT6B | DNM1 | GABRA1 | KIAA1468 | MSANTD3 | PDZD11 | RNMT | SURF2 | UGP2 |
| AR | CLIC1 | FAM89A | ICAM2 | MEGF10 | PHB | RSAD1 | STARD8 | WWOX |  | ANKRD39 | CD2BP2 | DNM3 | GABRG2 | KIF17 | MTCH1 | PEBP1 | ROBO1 | SUSD4 | UNC79 |
| ARAP1 | CLN3 | FANCE | ID3 | METTL7B | PHF10 | RUFY1 | STAT5B | XKR8 |  | ANKS1B | CD8A | DOC2A | GAD2 | KIF21A | MTHFD1 | PEX16 | ROPN1L | SV2A | UQCC1 |
| ARHGAP29 | CMTM3 | FAT1 | IER3 | MFNG | PHF2 | RUNX1 | STK11 | YAP1 |  | ANXA6 | CDC123 | DOLK | GALE | KIF24 | MTMR9 | PEX19 | RPA2 | SV2B | UQCR10 |
| ARHGAP4 | CNNM3 | FBLN1 | IFRD2 | MGAT1 | PHGDH | RUNX3 | STK38 | YBX1 |  | AP1AR | CDC27 | DOPEY1 | GALNT14 | KIN | MTR | PEX3 | RPAIN | SYBU | UQCRQ |
| ARHGAP9 | CNOT2 | FBRS | IGF2BP2 | MICALL2 | PIAS4 | RXRA | STON1 | YTHDF1 |  | AP2A2 | CDK14 | DPCD | GAP43 | KLHDC9 | MTX2 | PEX7 | RPL15 | SYNCRIP | USMG5 |
| ARHGEF26 | CNOT6 | FBXL7 | IL17RA | MKNK2 | PIGM | S100A10 | STX10 | ZBTB40 |  | AP3B2 | CDKN3 | DPH2 | GAPDH | KLHL26 | MYB | PFDN1 | RPS6KL1 | SYP | USP11 |
| ARHGEF40 | CNST | FBXO17 | IL17RB | MLXIP | PIK3CG | S100A4 | STXBP2 | ZBTB5 |  | APBB3 | CELF5 | DPP6 | GARNL3 | KLHL35 | MYH10 | PGAP3 | RRAGA | SYT13 | VAMP2 |
| ARPC1B | COL18A1 | FBXO18 | IL3RA | MMRN2 | PIM1 | SALL1 | STXBP4 | ZC2HC1C |  | APEX1 | CELF6 | DPP8 | GARS | KLK7 | MYL5 | PHYHIP | RSPH1 | SYT5 | VBP1 |
| ASXL1 | COL4A3BP | FBXO2 | IL4R | MOB3A | PKN2 | SAMD1 | SYNGR2 | ZC3H11A |  | APP | CENPF | DPY19L2 | GATB | KRT17 | MYO18B | PIAS2 | RTBDN | SYTL2 | VDAC2 |
| ATF1 | COL6A2 | FCGBP | ILK | MOB3C | PLAGL2 | SAMD11 | SYTL1 | ZC3H12A |  | ARC | CEP83 | DPYS | GFM1 | KRT222 | MYOZ3 | PIGB | RTCA | TAF9 | VPS4B |
| ATM | COL8A1 | FERMT3 | INPP5D | MOGAT1 | PLCD1 | SASH1 | SYTL4 | ZDHHC18 |  | AREL1 | CES4A | DRD5 | GLDC | KRT5 | NALCN | PIGZ | RTN3 | TAF9B | VRK1 |
| ATOH8 | COLGALT1 | FGFR4 | INPPL1 | MORC2 | PLCD3 | SCIN | TAF4 | ZFHX3 |  | ARF1 | CFAP36 | DRG1 | GLS | KTI12 | NAP1L2 | PIH1D2 | RTN4IP1 | TARBP1 | VSTM2L |
| ATP11C | CPM | FGL2 | IRAK1 | MORC4 | PLEKHA8P1 | SCRIB | TAP1 | ZFP36 |  | ARHGAP44 | CFAP46 | DTD1 | GMPR2 | LANCL1 | NAP1L3 | PIK3R4 | RUFY3 | TBC1D22A | VWC2 |
| ATP1B3 | CPSF1 | FKBP14 | IRF1 | MPST | PLEKHM1 | SDCCAG3 | TBC1D2B | ZHX1 |  | ARHGAP5-AS1 | CFC1 | DTNB | GNL3 | LAPTM4B | NAP1L5 | PITHD1 | RUNDC1 | TBCC | WASF1 |
| AUP1 | CRABP1 | FLII | IRF2BPL | MPZL2 | PLEKHO2 | SEC11A | TBL1X | ZNF175 |  | ARHGDIA | CHCHD3 | DUS2 | GOLGA8A | LARGE | NAPB | PJA2 | RWDD2A | TBP | WBP2 |
| B4GALT1 | CRB1 | FLNA | IRF3 | MRAS | PLOD1 | SEC61A1 | TBX3 | ZNF217 |  | ARNT2 | CHCHD7 | DUS4L | GOLT1A | LBH | NARS | PKD2L1 | RXFP1 | TCEA2 | WBP4 |
| BAG3 | CRB2 | FMO2 | IRF7 | MRPS6 | PNISR | SELO | TBX6 | ZNF219 |  | ASB2 | CHMP4B | DUSP11 | GOLT1B | LCMT1 | NAT16 | PKNOX2 | SATB2 | TCEAL2 | WDR47 |
| BANP | CSK | FOXC1 | ISG20 | MS4A7 | PNP | SEMA3F | TCAP | ZNF234 |  | ASIC2 | CHP1 | DUSP2 | GOT2 | LDOC1 | NAT6 | PLBD2 | SATB2-AS1 | TCEAL4 | WDR54 |
| BBX | CSRNP1 | FOXJ1 | IST1 | MSRB3 | PNPT1 | SERBP1 | TCF3 | ZNF358 |  | ATF7IP2 | CHRM1 | DUSP4 | GP1BB | LETMD1 | NDRG4 | PMS1 | SCFD2 | TCTEX1D1 | WDR61 |
| BCL2 | CTBP2 | FOXN3 | ISYNA1 | MSX1 | PODN | SERF2 | TCF7 | ZNF395 |  | ATP1A1 | CHSY3 | DYNC1H1 | GPI | LGALS8 | NDUFA1 | PMS2 | SCHIP1 | TERF2IP | WDR7 |
| BCL2L1 | CTDSP1 | FOXO1 | ITGA5 | MTA2 | POGK | SERPINA1 | TCF7L1 | ZNF438 |  | ATP1A3 | CIDEC | DYNC2LI1 | GPLD1 | LIN7B | NDUFA7 | PNMA2 | SDAD1 | TFB2M | WIF1 |
| BCL6 | CTRL | FRAT2 | ITGB5 | MTFP1 | POLD1 | SERPINB6 | TCIRG1 | ZNF524 |  | ATP1B1 | CIRBP | DYNLL1 | GPN2 | LINC00467 | NDUFB1 | PNMA5 | SEC16B | THOC3 | WNT10B |
| BCL6B | CTSD | FURIN | ITPR2 | MTMR3 | POLG | SERPINF2 | TEAD2 | ZNF561 |  | ATP5G1 | CITED1 | DYNLT3 | GPR22 | LINC01260 | NDUFB8 | PNMA6A | SEC61A2 | THYN1 | WRB |
| BHLHE41 | CWF19L2 | FYCO1 | ITPR3 | MTRF1 | POLH | SERPINH1 | TEAD4 | ZNF565 |  | ATP5J2 | CKMT1B | DZANK1 | GPR61 | LIPC | NDUFS5 | POLR2K | SEC61G | TIGD7 | XK |
| BMPR1A | CXCL1 | FYN | JDP2 | MUC1 | POLR2F | SERTAD1 | TEP1 | ZNF573 |  | ATP5O | CLCC1 | DZIP3 | GPRASP1 | LMO4 | NDUFV1 | POLR3B | SERINC1 | TIMM8A | YAE1D1 |
| BNIPL | CXCR4 | FZD7 | KAT6A | MVP | POLR2M | SH3TC1 | TES | ZNF587 |  | ATP6V0C | CLEC4G | EAPP | GPRASP2 | LOC440896 | NECAB1 | POLR3F | SETD9 | TIMM8B | YEATS4 |
| BOC | CYB561A3 | FZD9 | KCNJ10 | MXD4 | POMT2 | SIPA1 | TFPI | ZNF600 |  | ATP6V1F | CLTA | EBP | GREM2 | LOC81691 | NEFL | POMP | SEZ6L2 | TLK1 | YTHDF2 |
| BRAT1 | CYBA | GADD45G | KCNJ16 | MYBPH | POU2F2 | SIRT1 | TGFBI | ZNF646 |  | ATP6V1G1 | CLTC | EEF1E1 | GRM8 | LONRF2 | NEFM | POP4 | SH3GL2 | TLN2 | YWHAB |
| BRD4 | CYFIP1 | GALM | KDM5C | MYD88 | PPM1D | SIRT7 | TGFBR3 | ZNF672 |  | ATP6V1H | CLUL1 | EFCAB6 | GSS | LOR | NEU1 | PORCN | SH3RF1 | TM2D1 | YWHAG |
| BRWD3 | CYP1B1 | GAREML | KDM6A | MYH9 | PPP1R16A | SIX5 | TGIF2 | ZNF692 |  | ATPIF1 | CNIH3 | EGR4 | GTDC1 | LPPR1 | NEUROD6 | PPDPF | SIDT1 | TM6SF1 | ZBBX |
| BST2 | CYP2R1 | GAS1 | KIF1C | MYL12A | PPP4R1 | SLC12A4 | TIAL1 | ZNF768 |  | ATR | CNTNAP1 | EHD3 | GTF2H3 | LRFN5 | NFS1 | PPIH | SIPA1L2 | TM7SF2 | ZCCHC7 |
| C11orf30 | CYP39A1 | GATA2 | KLF2 | MYO1F | PRCP | SLC12A7 | TIMELESS | ZNF785 |  | ATRN | COG1 | EID2B | HACL1 | LRP11 | NGRN | PPL | SIRT3 | TMED4 | ZFP64 |
| C15orf52 | DAAM1 | GATAD2A | KLF6 | N4BP1 | PRELP | SLC12A9 | TIMP1 | ZNFX1 |  | AUH | COL9A3 | EIF1AX | HAGH | LRRC20 | NIF3L1 | PPME1 | SIRT5 | TMEFF2 | ZFPL1 |
| C19orf25 | DAPP1 | GCC1 | KLHL17 | N4BP2 | PRKD2 | SLC13A4 | TK1 | ZNRF3 |  | AUNIP | COPS7A | EIF1B | HARS | LRTOMT | NIPSNAP3B | PPOX | SLC10A4 | TMEM130 | ZKSCAN3 |
| C19orf44 | DBI | GCK | KLHL21 | NACC2 | PRKD3 | SLC15A3 | TLR5 |  |  | AZIN2 | COQ3 | EIF3K | HAS1 | LXN | NIT1 | PPP2R2B | SLC12A5 | TMEM141 | ZNF233 |
| C1orf64 | DCAF12 | GDF15 | KLKB1 | NCAPD2 | PRR14 | SLC18B1 | TMBIM1 |  |  | B3GALT2 | COQ4 | EIF4A2 | HBA1 | LYRM1 | NKAIN2 | PPP3CA | SLC22A17 | TMEM151A | ZNF236 |
| C1QA | DCLRE1C | GEM | KRT18 | NCF4 | PSD2 | SLC1A3 | TMEM106A |  |  | B3GALT6 | COX15 | ELAVL4 | HBD | LYRM9 | NKD2 | PRC1 | SLC22A18 | TMEM169 | ZNF25 |
| C1QTNF5 | DCN | GIMAP5 | KRT19 | NCOA3 | PSD4 | SLC1A5 | TMEM109 |  |  | B3GNT4 | COX16 | ELMO1 | HDDC3 | LZTS1 | NLRP3 | PRDM2 | SLC25A25 | TMEM178A | ZNF334 |
| C21orf62 | DDAH2 | GIMAP8 | LAMA2 | NDUFA4L2 | PSMB8 | SLC22A23 | TMEM119 |  |  | B9D1 | COX18 | ELOVL4 | HECTD2 | M6PR | NOL4L | PRDX2 | SLC25A26 | TMEM191A | ZNF529 |
| C3 | DDIT4L | GJA1 | LAMA5 | NEK4 | PTAR1 | SLC22A6 | TMEM120A |  |  | BASP1 | COX4I1 | ELOVL6 | HEY1 | MAD2L1 | NOMO1 | PREP | SLC25A33 | TMEM30A | ZNF534 |
| C4A | DDR2 | GJA4 | LAMB2 | NEO1 | PTBP1 | SLC25A1 | TMEM156 |  |  | BCKDHB | COX6A1 | EMC4 | HIGD1A | MAEL | NOMO2 | PRKAG1 | SLC25A5 | TMEM50A | ZNF706 |
| C7 | DDX23 | GKAP1 | LAMC1 | NEXN | PTCHD1 | SLC26A1 | TMEM189 |  |  | BCL2L2 | COX6B1 | EMC7 | HIVEP1 | MAGEE1 | NOS2 | PRMT9 | SLC39A3 | TMLHE | ZNF85 |
| C9orf142 | DDX39A | GLI2 | LAPTM5 | NFAT5 | PTH1R | SLC38A2 | TMEM216 |  |  | BDH1 | COX7A2 | ENPP5 | HK1 | MAGI3 | NOVA1 | PRPF4 | SLC39A9 | TMX3 | ZSCAN1 |
| CACFD1 | DEK | GNA13 | LAT2 | NFATC1 | PTTG1IP | SLC39A1 | TMX1 |  |  | BEND5 | COX7A2L | ENY2 | HMGB3 | MAK16 | NOXA1 | PRR16 | SLC9A6 | TNNI3K | ZSCAN18 |
| CADM1 | DHCR7 | GNAI2 | LCAT | NFATC3 | PXDC1 | SLC39A11 | TNFAIP8 |  |  | BFSP1 | COX7B | EPB41L3 | HMGN2 | MANBAL | NPB | PRR4 | SMAP2 | TOX2 | ZSCAN5A |
| CARD6 | DLC1 | GNB2L1 | LDLR | NFIA | RAB27A | SLC39A12 | TNFRSF10B |  |  | BHLHB9 | CPNE4 | EPB41L4B | HNRNPH2 | MANSC1 | NPM2 | PRSS16 | SMS | TOX4 |  |
| CARS2 | DLEU1 | GOLGA6L5P | LDLRAD3 | NFIC | RAB29 | SLC3A2 | TNFRSF11B |  |  | BLCAP | CPNE5 | EPCAM | HOMER2 | MAP10 | NRBP2 | PRSS35 | SMYD2 | TPRG1L |  |
| CASKIN2 | DLG5 | GOLIM4 | LEAP2 | NFKB1 | RAB3IL1 | SLC43A3 | TNFRSF12A |  |  | BNIP3 | CPNE7 | EPS15 | HOPX | MAP7D2 | NRCAM | PSEN2 | SNCA | TRAM1L1 |  |
| CASP4 | DNAJC1 | GPER1 | LEMD2 | NFKBIZ | RAD54L2 | SLC52A3 | TNFRSF1A |  |  | BPHL | CREG2 | ERCC2 | HRH1 | MAPK6 | NRN1 | PSMA3 | SNRNP25 | TRAP1 |  |
| CASP7 | DNHD1 | GPR4 | LHFPL2 | NGLY1 | RAMP3 | SLC5A3 | TNFRSF1B |  |  | BRD9 | CRY2 | EXOC8 | HSBP1 | MARCKSL1 | NT5DC3 | PSMB4 | SNRPB2 | TRAPPC1 |  |
| CC2D1A | DNM2 | GPRC5C | LIFR | NHLRC2 | RASEF | SLC6A12 | TNFRSF6B |  |  | BRE | CSMD1 | EXOSC6 | HSD11B1L | MBOAT2 | NT5E | PSMD10 | SNRPD1 | TRAPPC11 |  |

**Supplementary Table 3. Characteristics of animal models of AD**

| Models | Model classification | Public  Sources* | Genes | Status of genes | Conditions | Reference |
| --- | --- | --- | --- | --- | --- | --- |
| KNL-HYD-TG0610  (NCSTN model) | Transgenic | - | NCSTN | Mutant type |  | [10, 6] |
| KNL-HYD-TG0606  (PSEN2 model) | Transgenic | - | PSEN2 | Mutant type |  | [9] |
| KNL-HYD-TG0601  (MAPT model) | Transgenic | - | MAPT | Wild type |  | [19] |
| 5×FAD | Transgenic | - | APP, PSEN1 | Mutant type |  | [18, 13] |
| Aged mice | Ageing model | - | - | - | 1.5, 4, 9, 17, and 22 months old |  |
| BCCAO | Non-transgenic | - | - | - | Surgical induction of bilateral common carotid artery occlusion (BCCAO)  (14 ~ 70 days after surgery) | [1] |
| Aβ injection | Non-transgenic | - | - | - | i.c.v. injection of Aβ  (5 and 10 µM) | [12] |
| Streptozotocin model | Pharmacological | - | - | - | i.c.v. injection of streptozotocin  (2.5 and 3 mg/kg) | [3] |
| Scopolamine  model | Pharmacological | - | - | - | i.p. injection of scopolamine  (1 mg/kg) | [7] |
| TgCRND8 mice | Transgenic | GSE31372 | APP | Mutant type |  | [4] |
| 3×Tg-AD-H mice  3×Tg-AD-h mice | Transgenic | GSE36981 | APP, PSEN1, MAPT | Mutant type | 3×Tg-AD-H mice:  Homozygous mutant transgenes for APPSwe and tauP301L  3×Tg-AD-h mice:  Hemizygous mutant transgenes for APPSwe and tauP301L | [8] |
| 3×Tg/WT Polβ  3×Tg/Polβ(+/-) | Transgenic | GSE60911 | APP, PSEN1, MAPT, POLB | Mutant type |  | [20] |
| APP23 mice | Transgenic | GSE80465 | APP | Mutant type |  | [11] |

^*^Information pertaining to microarrays was deposited in Gene Expression Omnibus (http://www.ncbi.nlm.nih.gov/geo)

**Supplementary references**

1. Bang J, Jeon WK, Lee IS, Han JS, Kim BY. Biphasic functional regulation in hippocampus of rat with chronic cerebral hypoperfusion induced by permanent occlusion of bilateral common carotid artery. PLoS One. 2013; doi:10.1371/journal.pone.0070093.
2. Berchtold NC, Coleman PD, Cribbs DH, Rogers J, Gillen DL, Cotman CW. Synaptic genes are extensively downregulated across multiple brain regions in normal human aging and Alzheimer's disease. Neurobiol Aging. 2013;34:1653–61.
3. Chen Y, Liang Z, Blanchard J, Dai CL, Sun S, Lee MH, Grundke-Iqbal I, Iqbal K, Liu F, Gong CX. A non-transgenic mouse model (icv-STZ mouse) of Alzheimer's disease: similarities to and differences from the transgenic model (3xTg-AD mouse). Mol Neurobiol. 2013; 47:711–25.
4. Chishti MA, Yang DS, Janus C, Phinney AL, Horne P, Pearson J, Strome R, Zuker N, Loukides J, French J, Turner S, Lozza G, Grilli M, Kunicki S, Morissette C, Paquette J, Gervais F, Bergeron C, Fraser PE, Carlson GA, George-Hyslop PS, Westaway D. Early-onset amyloid deposition and cognitive deficits in transgenic mice expressing a double mutant form of amyloid precursor protein 695. J Biol Chem. 2001;276:21562–70.
5. Colantuoni C, Lipska BK, Ye T, Hyde TM, Tao R, Leek JT, Colantuoni EA, Elkahloun AG, Herman MM, Weinberger DR, Kleinman JE. Temporal dynamics and genetic control of transcription in the human prefrontal cortex. Nature. 2011;478:519–23.
6. Confaloni A, Terreni L, Piscopo P, Crestini A, Campeggi LM, Frigerio CS, Blotta I, Perri M, Di Natale M, Maletta R, Marcon G, Franceschi M, Bruni AC, Forloni G, Cantafora A. Nicastrin gene in familial and sporadic Alzheimer's disease. Neurosci Lett. 2003;353:61–5.
7. Gilles C, Ertlé S. Pharmacological models in Alzheimer's disease research. Dialogues Clin Neurosci. 2000;2:247–55.
8. Hokama M, Oka S, Leon J, Ninomiya T, Honda H, Sasaki K, Iwaki T, Ohara T, Sasaki T, LaFerla FM, Kiyohara Y, Nakabeppu Y. Altered expression of diabetes-related genes in Alzheimer's disease brains: the Hisayama study. Cereb Cortex. 2014;24:2476–88.
9. Hwang DY, Chae KR, Kang TS, Hwang JH, Lim CH, Kang HK, Goo JS, Lee MR, Lim HJ, Min SH, Cho JY, Hong JT, Song CW, Paik SG, Cho JS, Kim YK. Alterations in behavior, amyloid beta-42, caspase-3, and Cox-2 in mutant PS2 transgenic mouse model of Alzheimer's disease. FASEB J. 2002;16:805–13.
10. Hwang DY, Kim YK, Lim CJ, Cho JS. Mutant nicastrin protein can induce the cytochrome c release and the Bax expression. Int J Neurosci. 2004;114:1277–89.
11. Janssen L, Dubbelaar ML, Holtman IR, de Boer-Bergsma J, Eggen BJ, Boddeke HW, De Deyn PP, Van Dam D. Aging, microglia and cytoskeletal regulation are key factors in the pathological evolution of the APP23 mouse model for Alzheimer's disease. Biochim Biophys Acta. 2017; 1863:395–405.
12. Kim HY, Kim HV, Jo S, Lee CJ, Choi SY, Kim DJ, Kim Y. EPPS rescues hippocampus-dependent cognitive deficits in APP/PS1 mice by disaggregation of amyloid-β oligomers and plaques. Nat Commun. 2015; doi:10.1038/ncomms9997.
13. Kimura R, Ohno M. Impairments in remote memory stabilization precede hippocampal synaptic and cognitive failures in 5XFAD Alzheimer mouse model. Neurobiol Dis. 2009;33:229–35.
14. Liang WS, Reiman EM, Valla J, Dunckley T, Beach TG, Grover A, Niedzielko TL, Schneider LE, Mastroeni D, Caselli R, Kukull W, Morris JC, Hulette CM, Schmechel D, Rogers J, Stephan DA. Alzheimer's disease is associated with reduced expression of energy metabolism genes in posterior cingulate neurons. Proc Natl Acad Sci U S A. 2008;105:4441–6.
15. Lu T, Pan Y, Kao SY, Li C, Kohane I, Chan J, Yankner BA. Gene regulation and DNA damage in the ageing human brain. Nature. 2004;429:883–91.
16. Lu T, Aron L, Zullo J, Pan Y, Kim H, Chen Y, Yang TH, Kim HM, Drake D, Liu XS, Bennett DA, Colaiácovo MP, Yankner BA. REST and stress resistance in ageing and Alzheimer's disease. Nature. 2014;507:448–54.
17. Narayanan M, Huynh JL, Wang K, Yang X, Yoo S, McElwee J, Zhang B, Zhang C, Lamb JR, Xie T, Suver C, Molony C, Melquist S, Johnson AD, Fan G, Stone DJ, Schadt EE, Casaccia P, Emilsson V, Zhu J. Common dysregulation network in the human prefrontal cortex underlies two neurodegenerative diseases. Mol Syst Biol. 2014; doi:10.15252/msb.20145304.
18. Oakley H, Cole SL, Logan S, Maus E, Shao P, Craft J, Guillozet-Bongaarts A, Ohno M, Disterhoft J, Van Eldik L, Berry R, Vassar R. Intraneuronal beta-amyloid aggregates, neurodegeneration, and neuron loss in transgenic mice with five familial Alzheimer's disease mutations: potential factors in amyloid plaque formation. J Neurosci. 2006;26:10129–40.
19. Shim SB, Lim HJ, Chae KR, Kim CK, Hwang DY, Jee SW, Lee SH, Sin JS, Leem YH, Lee SH, Cho JS, Lee HH, Choi SY, Kim YK. Tau overexpression in transgenic mice induces glycogen synthase kinase 3beta and beta-catenin phosphorylation. Neuroscience. 2007;146:730–40.
20. Sykora P, Misiak M, Wang Y, Ghosh S, Leandro GS, Liu D, Tian J1, Baptiste BA, Cong WN, Brenerman BM, Fang E, Becker KG, Hamilton RJ, Chigurupati S, Zhang Y, Egan JM, Croteau DL, Wilson DM 3rd, Mattson MP, Bohr VA. DNA polymerase β deficiency leads to neurodegeneration and exacerbates Alzheimer disease phenotypes. Nucleic Acids Res. 2015;43:943–59.
21. Webster JA, Gibbs JR, Clarke J, Ray M, Zhang W, Holmans P, Rohrer K, Zhao A, Marlowe L, Kaleem M, McCorquodale DS 3rd, Cuello C, Leung D, Bryden L, Nath P, Zismann VL, Joshipura K, Huentelman MJ, Hu-Lince D, Coon KD, Craig DW, Pearson JV; NACC-Neuropathology Group, Heward CB, Reiman EM, Stephan D, Hardy J, Myers AJ. Genetic control of human brain transcript expression in Alzheimer disease. Am J Hum Genet. 2009;84:445–58.
22. Zhang B, Gaiteri C, Bodea LG, Wang Z, McElwee J, Podtelezhnikov AA, Zhang C, Xie T, Tran L, Dobrin R, Fluder E, Clurman B, Melquist S, Narayanan M, Suver C, Shah H, Mahajan M, Gillis T, Mysore J, MacDonald ME, Lamb JR, Bennett DA, Molony C, Stone DJ, Gudnason V, Myers AJ, Schadt EE, Neumann H, Zhu J, Emilsson V. Integrated systems approach identifies genetic nodes and networks in late-onset Alzheimer's disease. Cell. 2013;153:707–20.


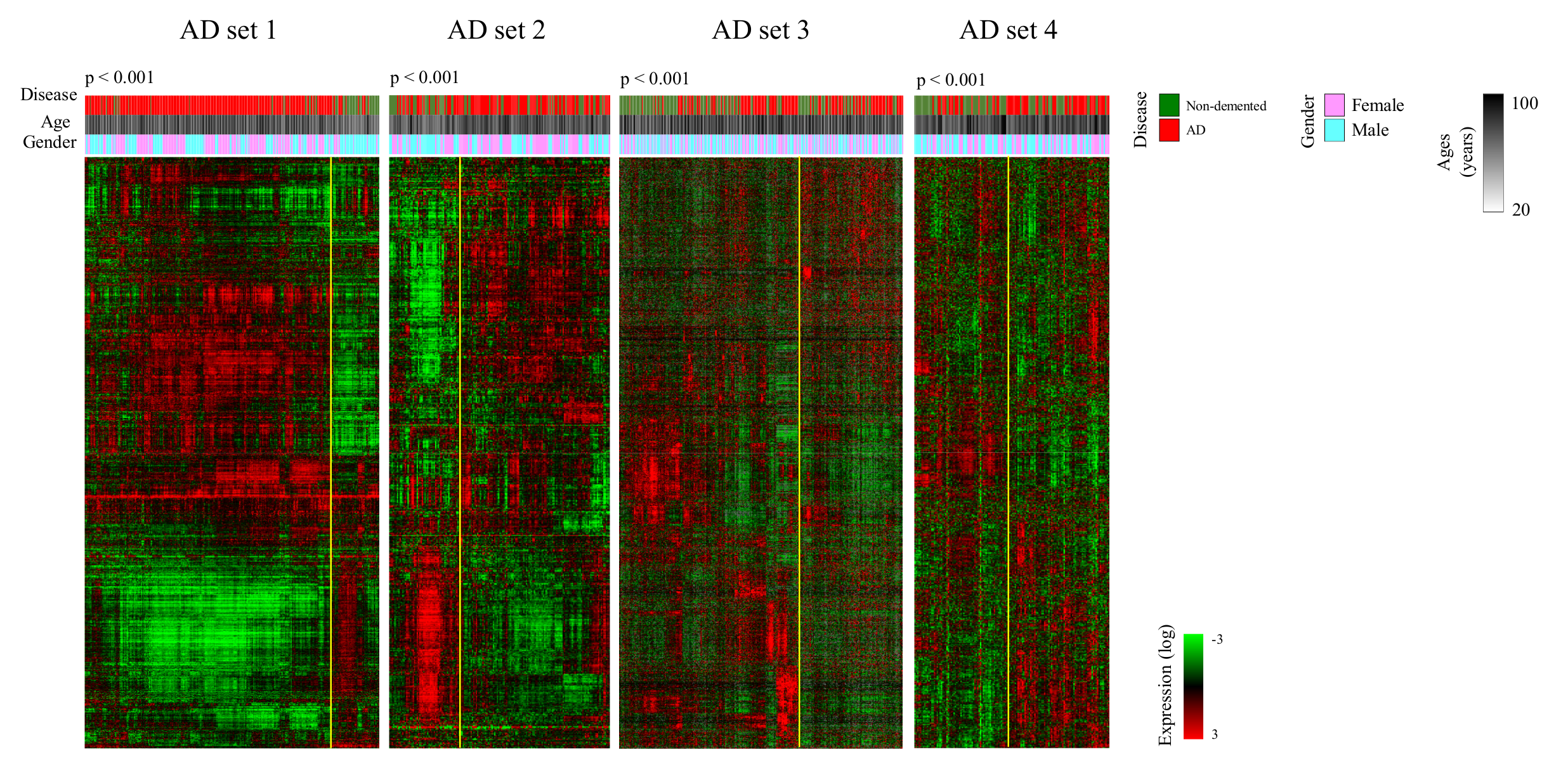


**Supplementary Fig. 1.** Clustering patterns of gene expression exclusively in samples of individuals aged >65 years from each AD set. Genes that showed a variation over 0.25 SD in the expression levels of genes in each dataset were selected and hierarchically clustered. Columns represent individual samples and rows represent genes. Red and green colors reflect high and low expression levels, respectively, as indicated by the scale bars. In the tree structure, green and red samples represent nondemented and AD individuals, respectively. Two subgroups of samples in each dataset were discriminated with yellow lines. Differential distribution of AD and nondemented individuals in two subgroups (p < 0.001) was measured using chi-square test with Yates’s continuity correction.


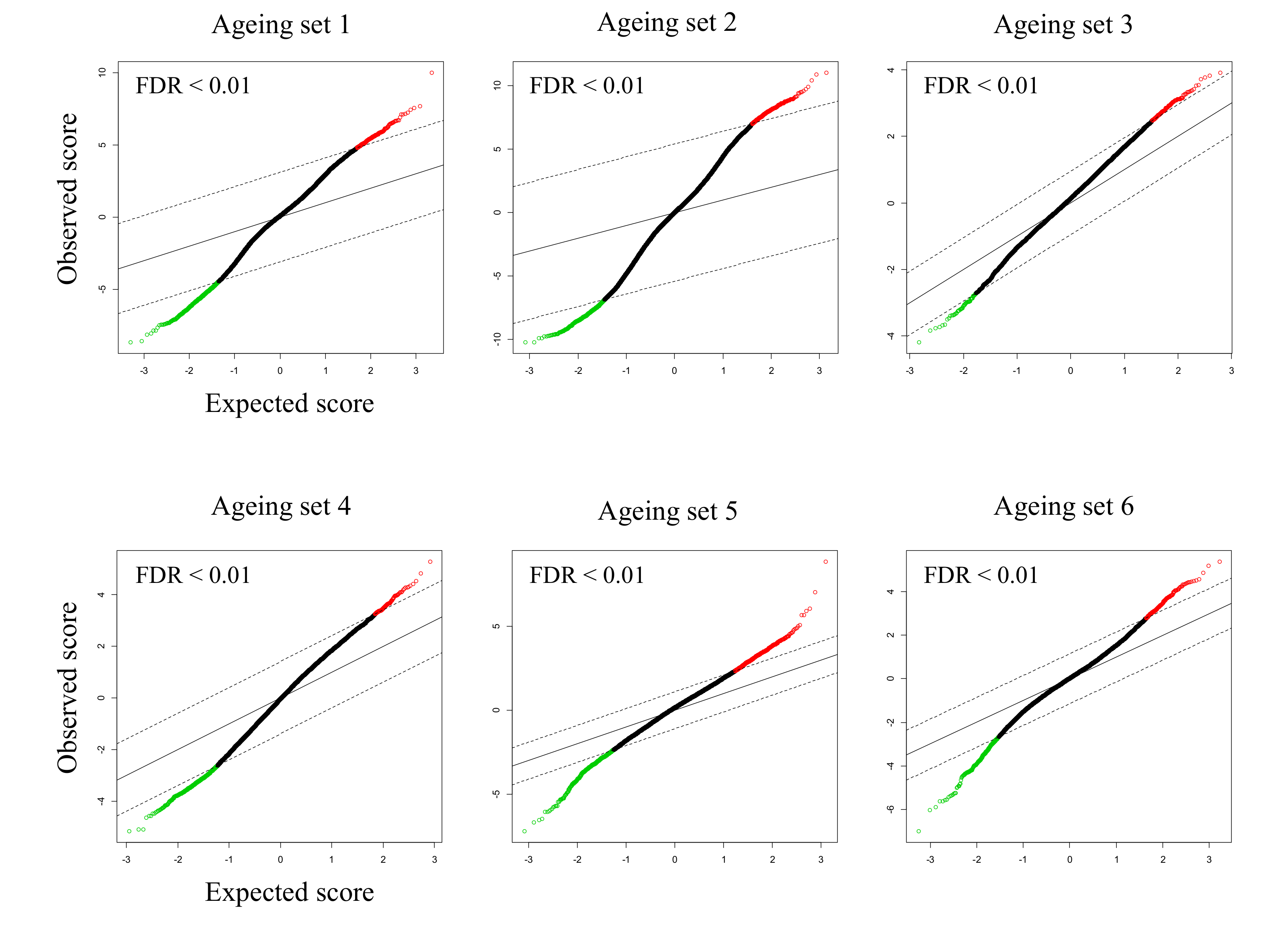


**Supplementary Fig. 2.** Genes associated with ageing were identified using Significance Analysis of Microarray (SAM). For the microarray data from four datasets, SAM scores were used for the threshold value corresponding to an FDR < 0.01. Red and green circles represent genes in the AD samples that were up- and downregulated, respectively, compared with nondemented control samples. The dotted line represents the threshold line.


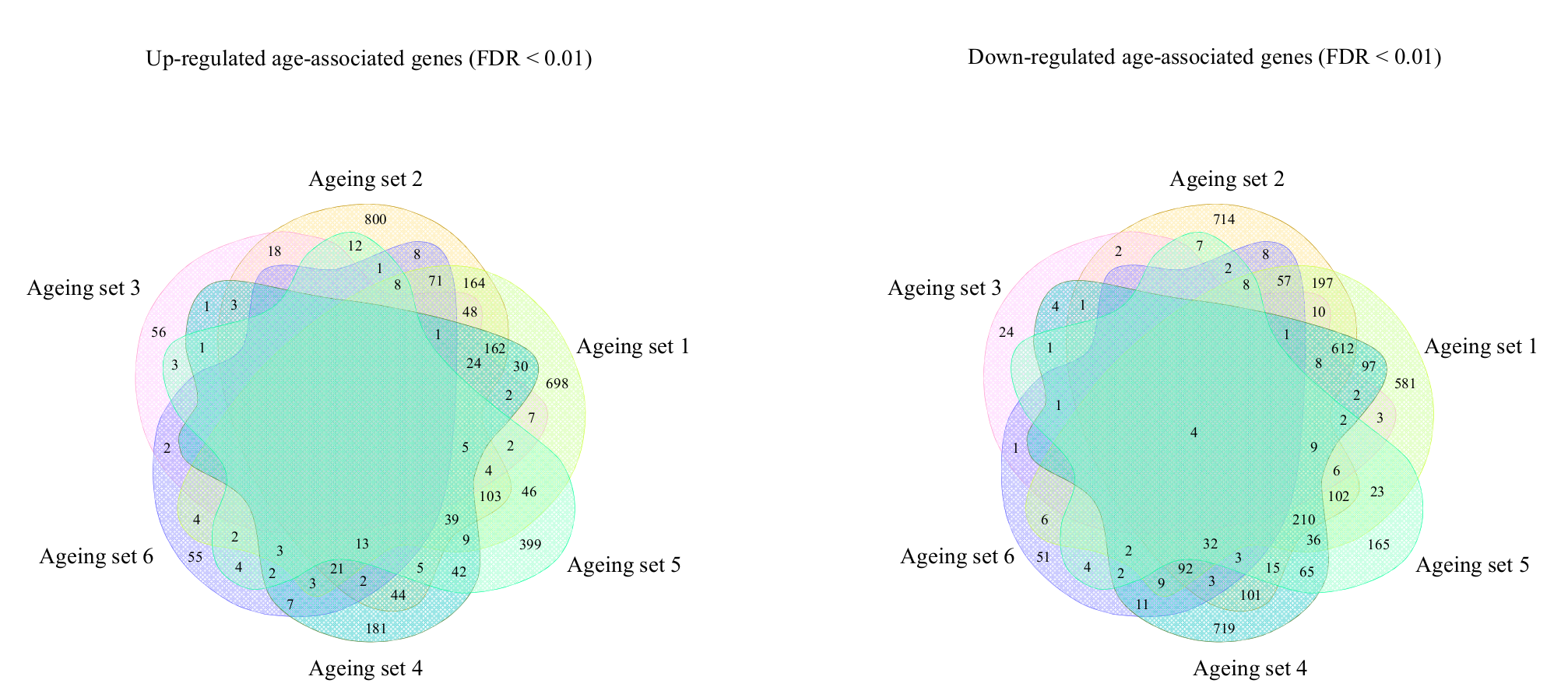


**Supplementary Fig. 3.** Distribution of age-associated genes (FDR < 0.01) from six datasets.


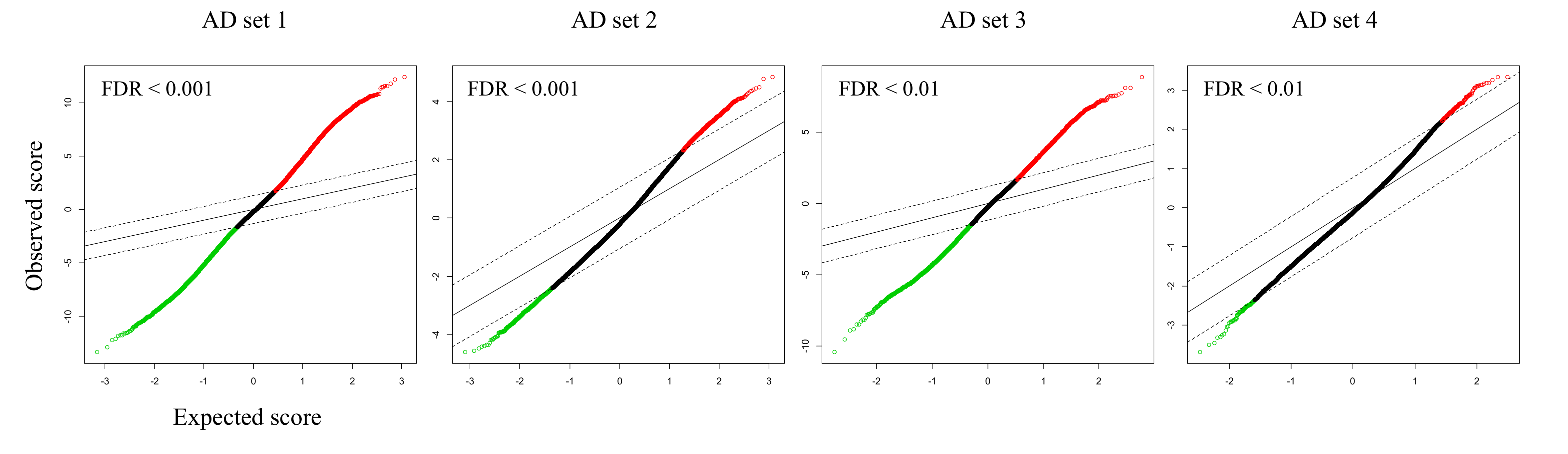


**Supplementary Fig. 4.** Genes associated with AD were identified using Significance Analysis of Microarray (SAM). For the microarray data from four datasets, SAM scores were used for the threshold value corresponding to an FDR < 0.01. For AD sets 1 and 2, the threshold value was set more stringently at FDR < 0.001. Red and green circles represent genes that were up- and downregulated in the AD samples, respectively, compared with nondemented control samples. The dotted line represents the threshold line.


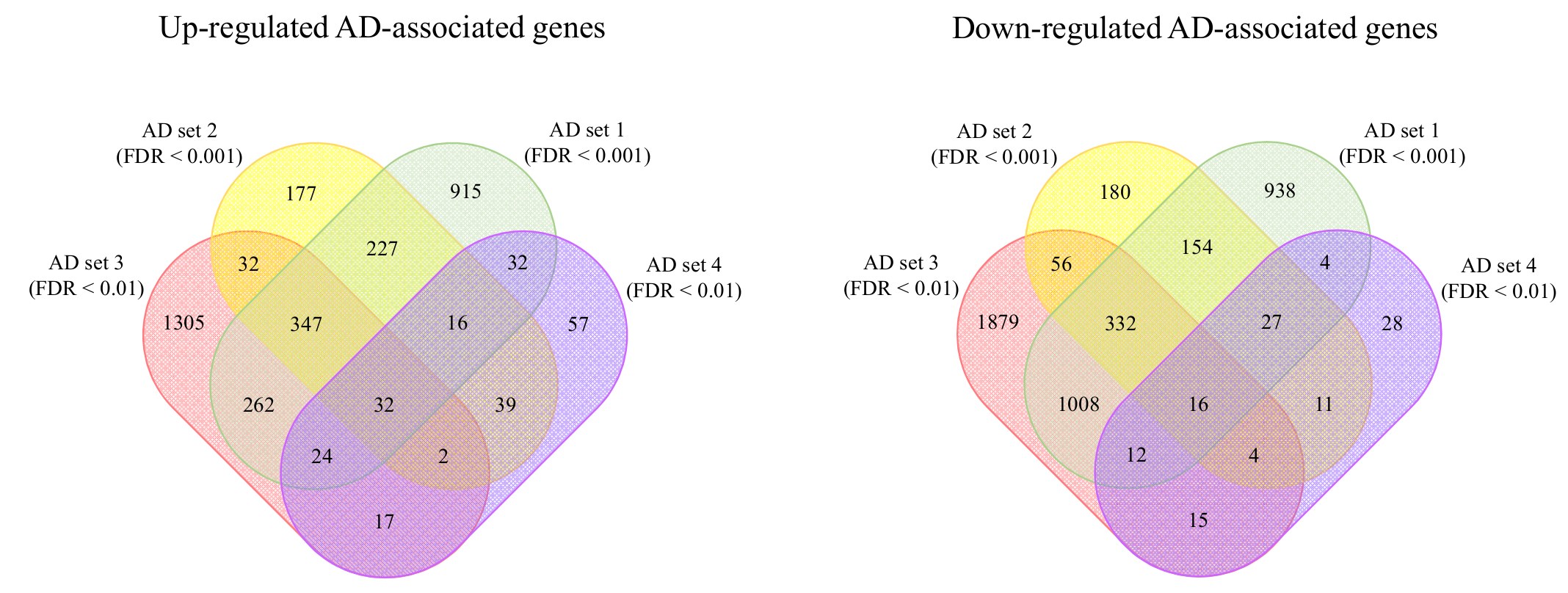


**Supplementary Fig. 5.** Distribution of AD-associated genes from four datasets.


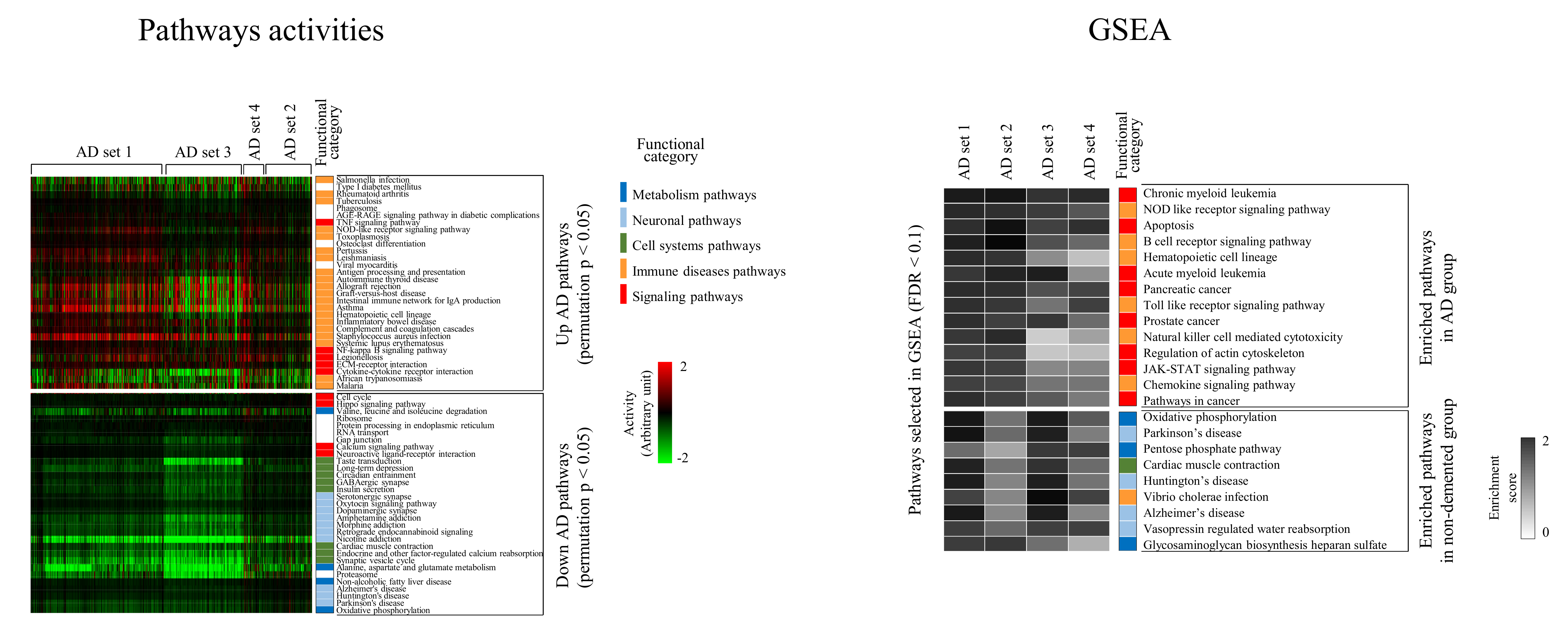


**Supplementary Fig. 6.** Pathways that were differentially activated or differentially enriched between AD and nondemented individuals (permutation p < 0.05) were selected and their activities or enrichment scores were visualized using a color scale. Columns represent individual samples and rows represent pathways. The functional categories of pathways (FDR < 0.1) are displayed in color bars. Enrichment scores for each pathway are indicated as black and white intensity, as shown in the scale bar.


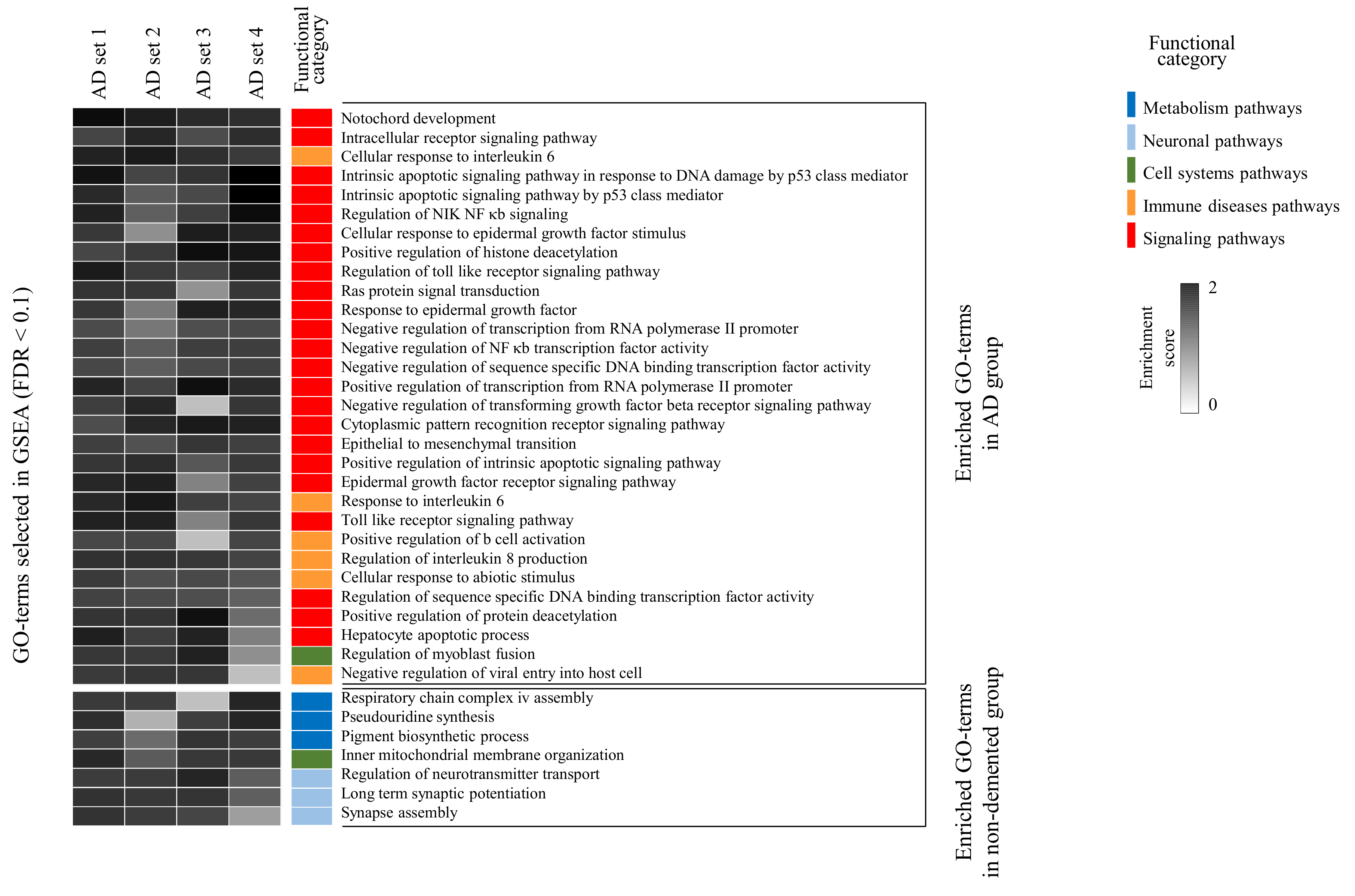


**Supplementary Fig. 7.** GO terms (FDR < 0.1) that were enriched commonly in all datasets were selected from GSEA. The functional categories of GO terms are displayed in color bars. Enrichment scores for each GO term are indicated in black and white intensity, as shown in the scale bar.


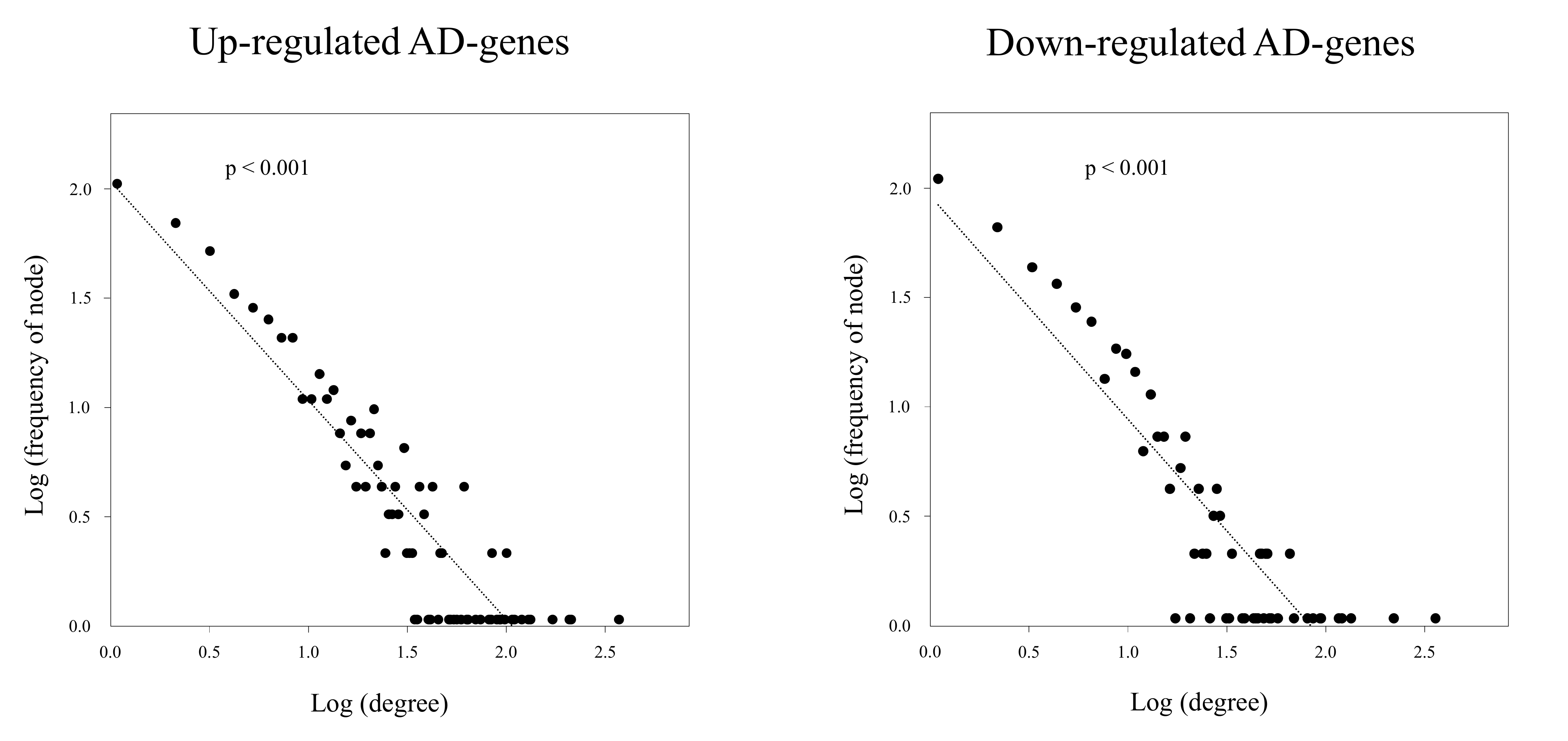


**Supplementary Fig. 8.** Plot of node frequencies according to degrees of nodes. Up- or downregulated AD-genes were used as nodes. The distribution of the node degrees of genes was measured in the protein–protein interaction (PPI) network obtained from the database of BioGRID (version 3.4.149).


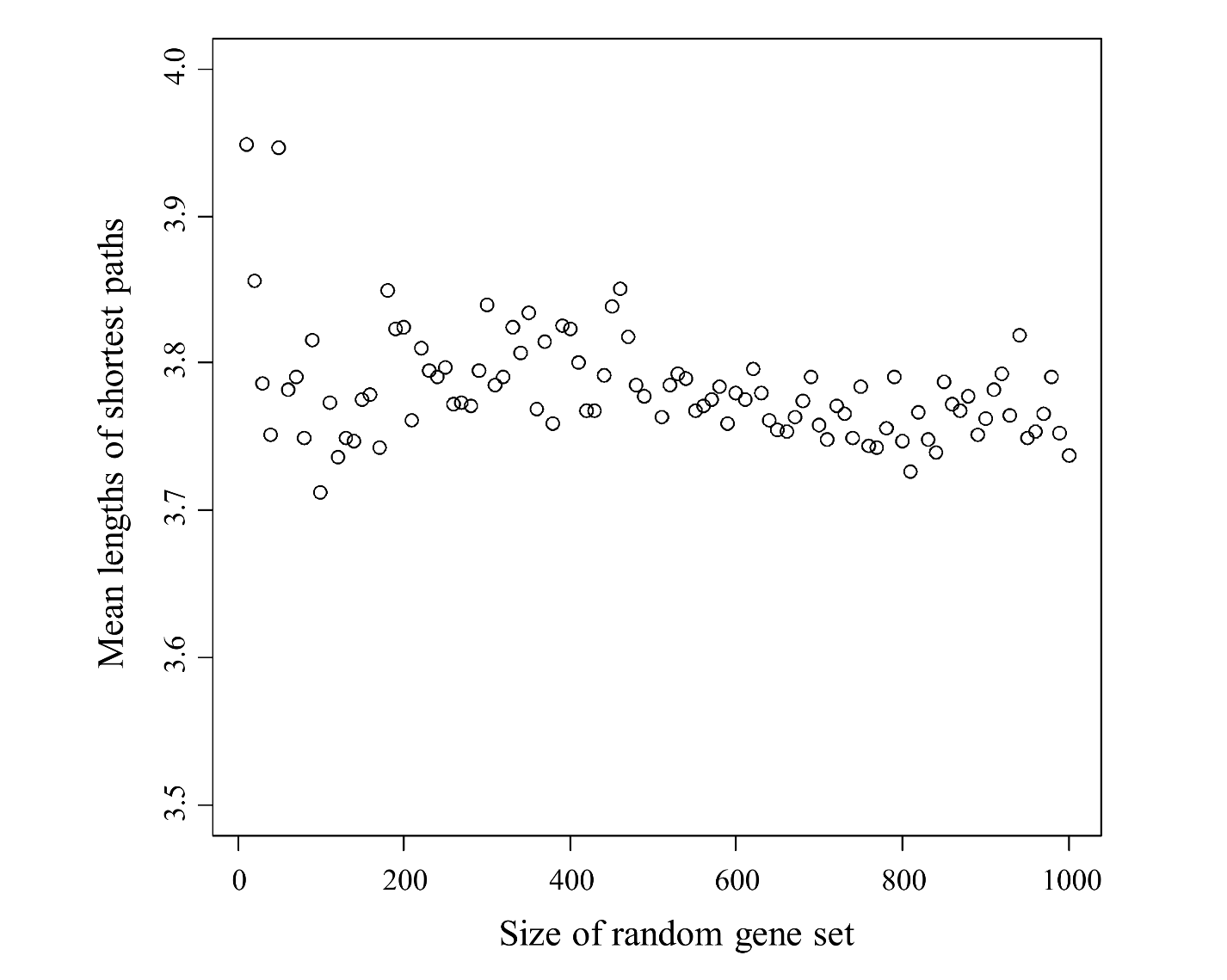


**Supplementary Fig. 9.** Effects of the size of gene sets on distances in the PPI network. The length of the shortest paths was measured between two randomly selected gene sets by varying the number of genes.

**
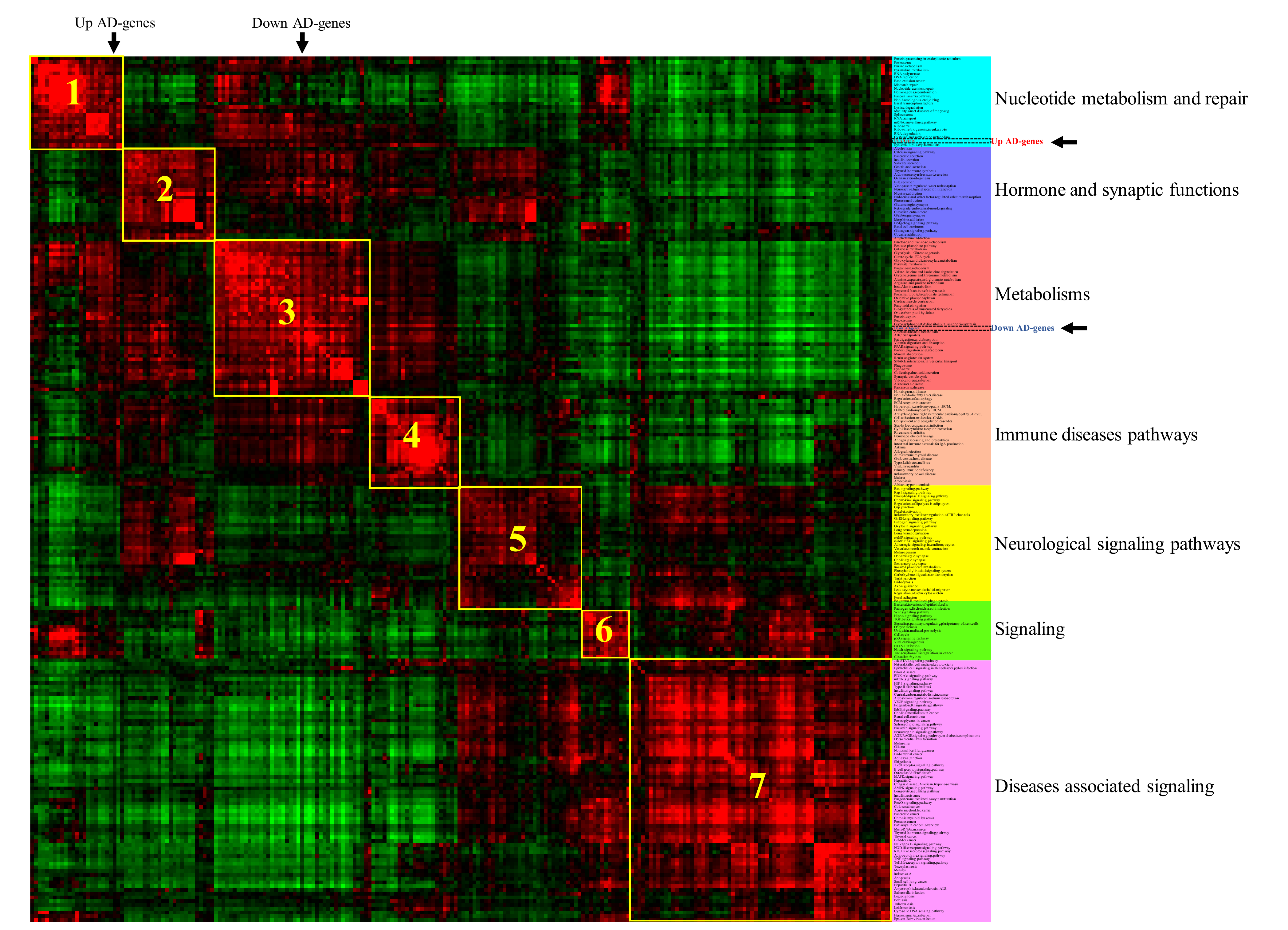
**

**Supplementary Fig. 10.** Clustering of pathways based on distances in the PPI network. For the measurement of distances between two gene sets, each gene was mapped onto the PPI network. Subsequently, the shortest paths between two genes were measured using the igraph R package (version 1.0.10). After iteration of this process for all pairs of genes from two gene sets, we obtained a matrix of shortest paths. The averaged value from the matrix of shortest paths is presented as the distance between two sets of genes. Only sets of genes with a presence in the PPI network >50% were included. The effect of the size of the gene set was measured using a random-permutation-based approach (iteration number, 1,000), in which distances were measured between random-sized gene sets. Clusters of pathways were numbered according to representative biological functions. Individual pathways included in each cluster are also indicated.


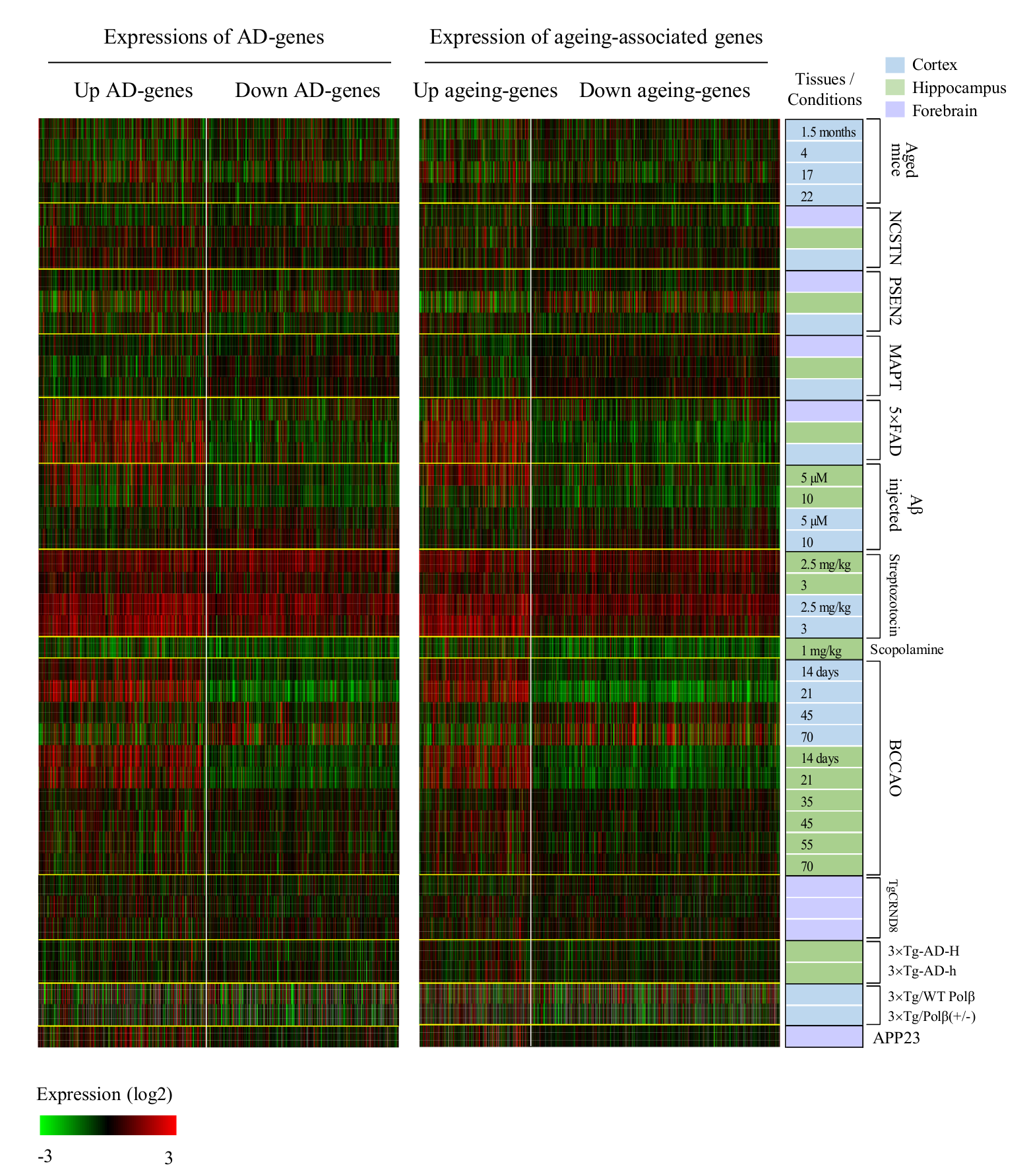


**Supplementary Fig. 11.** Expression profile of AD-genes and age-associated genes in animal models. The expression levels of AD-genes or age-associated genes in animal models were compared quantitatively. Red and blue bars indicate averaged values obtained using upregulated AD-genes (or upregulated age-associated genes) and downregulated AD-genes (or downregulated age-associated genes), respectively.

**
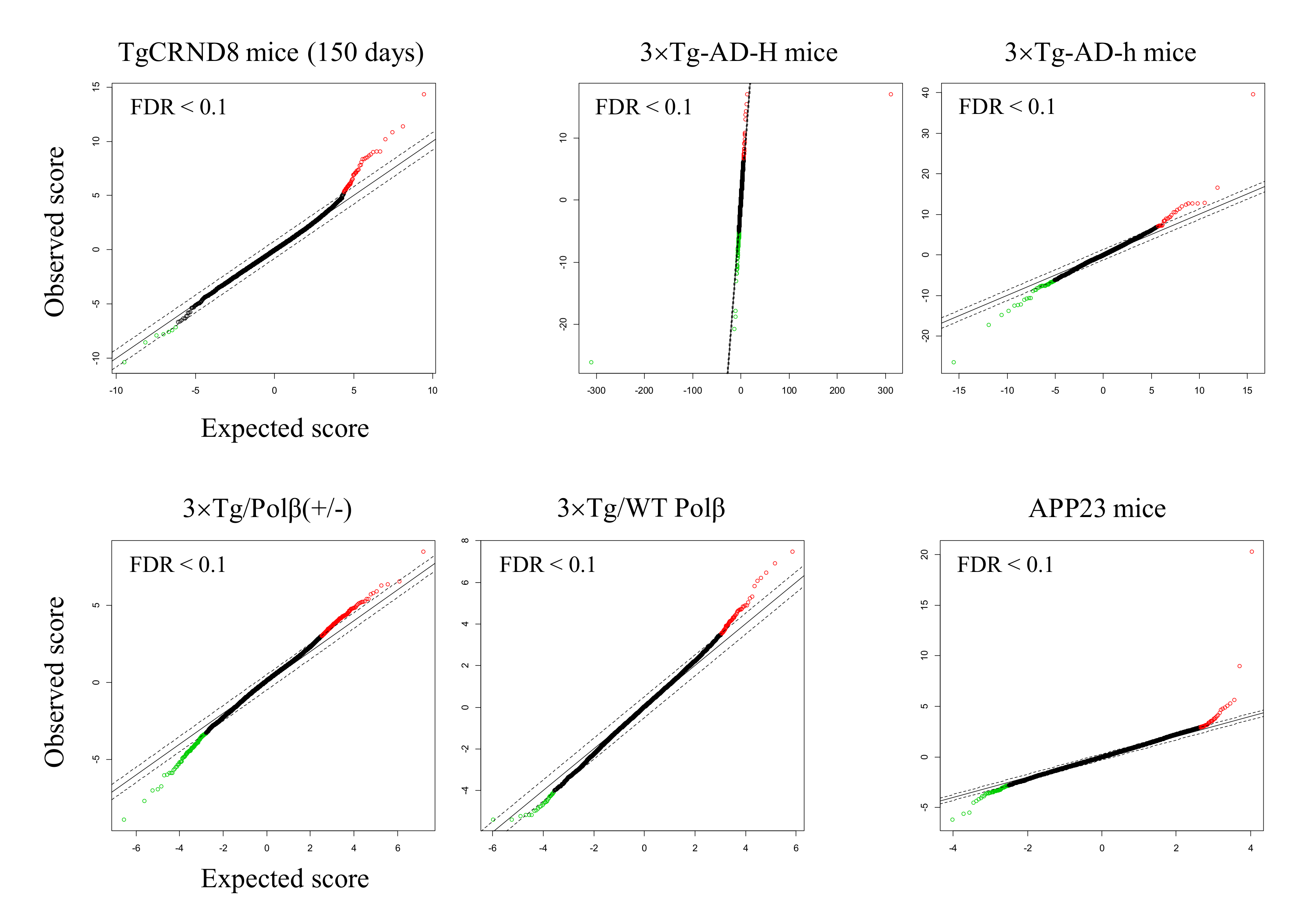
**

**Supplementary Fig. 12.** Differentially expressed genes (DEGs) from each animal model were identified using Significance Analysis of Microarray (SAM). FDR < 0.1 under the two-class response type option of SAM was used as a selection criteria for DEGs. Red and green circles represent genes that were up- and downregulated, respectively, compared with control samples. The dotted line represents the threshold line.


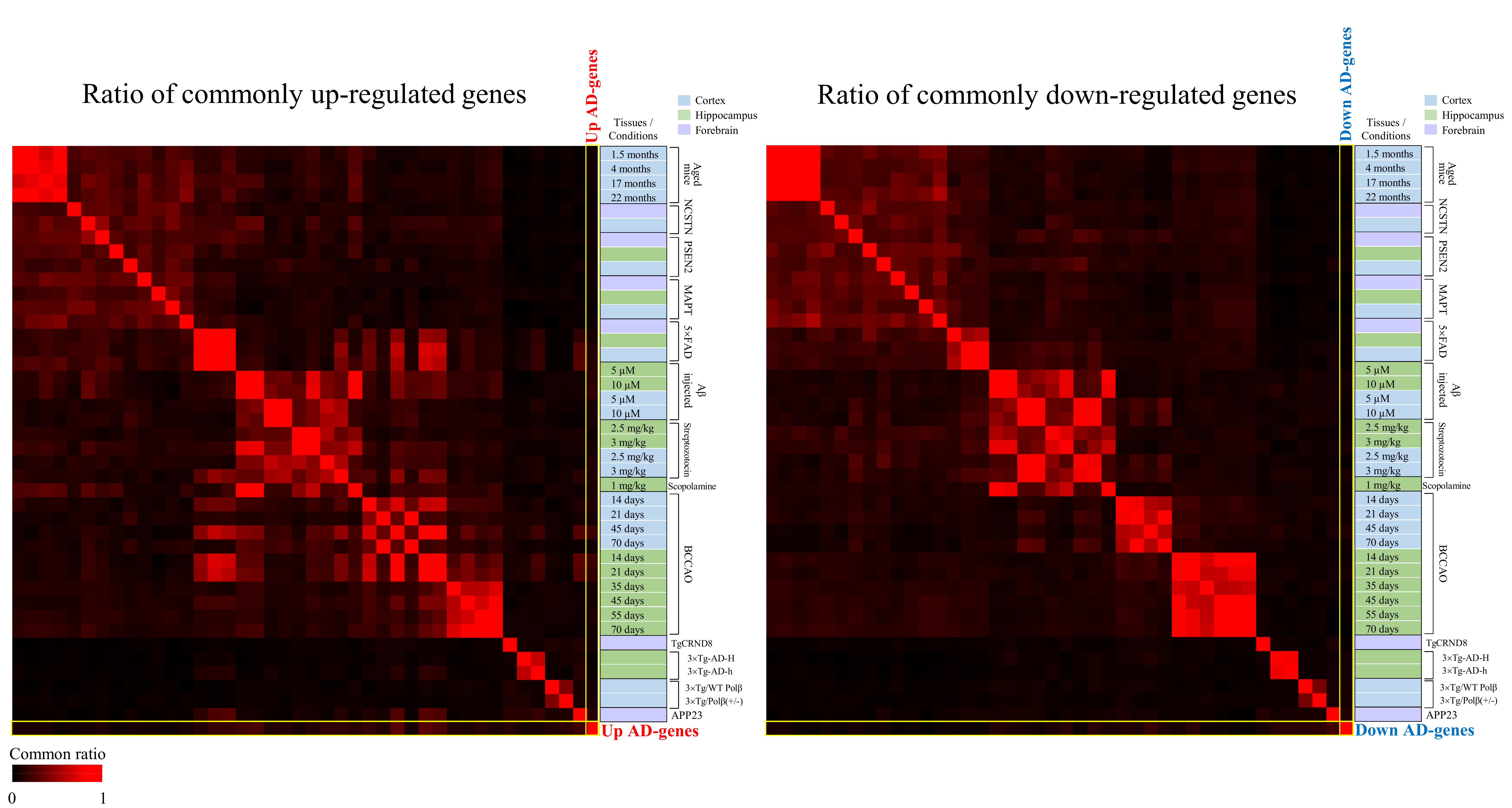


**Supplementary Fig. 13.** Ratio of common genes between animal models. The number of common genes between differentially expressed genes (DEGs) from animal models and AD-genes was compared. DEGs were selected using a 2-fold criterion for the animal models used here, whereas FDR < 0.1 under the two-class response type option of SAM was used as a selection criterion for DEGs from animal models from the GEO database (TgCRND8, 3×Tg, and APP23 models)


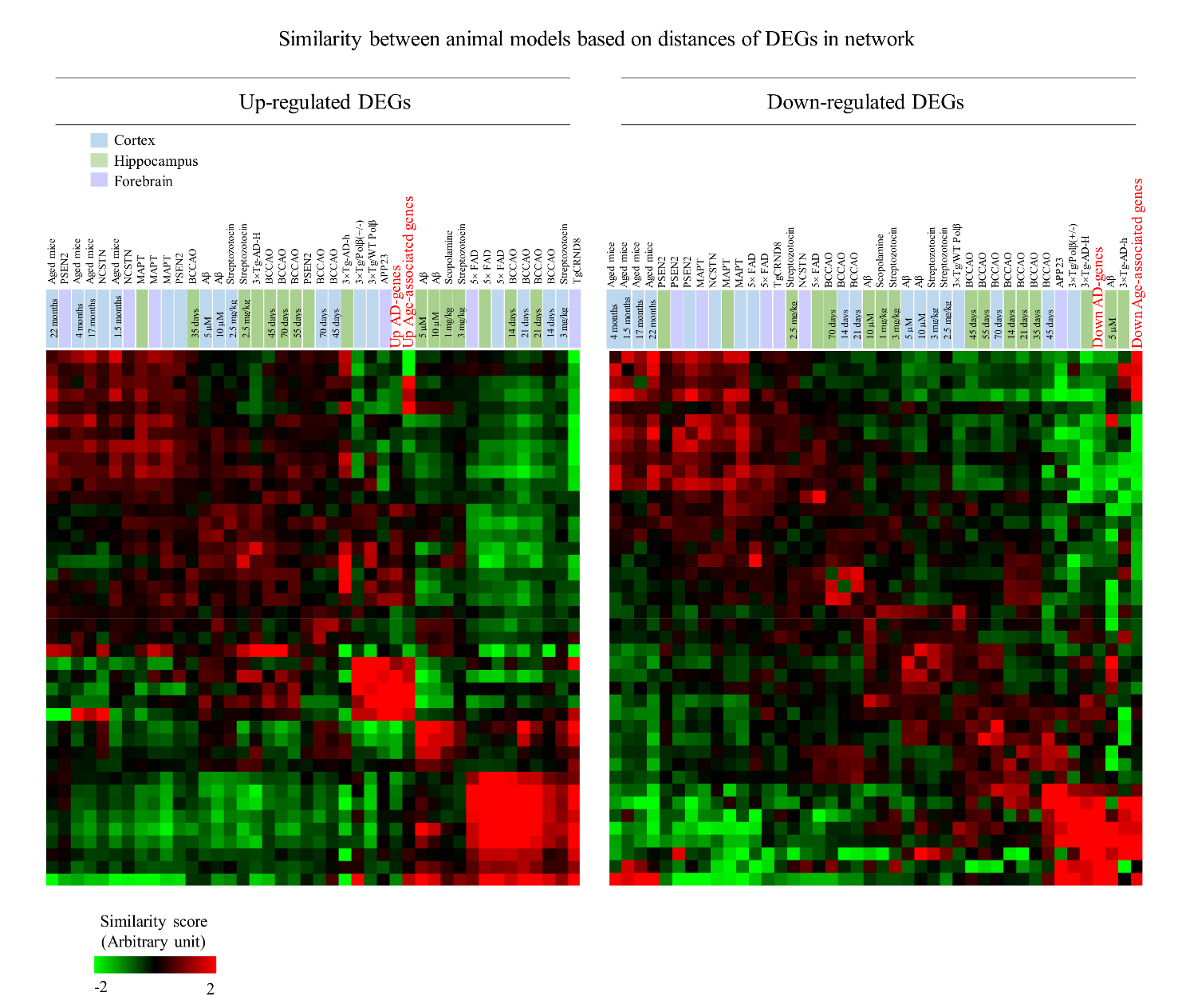


**Supplementary Fig. 14.** The similarity between age-associated genes and AD-genes was examined by measuring the clustering patterns of distances to the DEGS of animal models. The positions of up- and downregulated AD-genes (or age-associated genes) are highlighted in red color.
